# Prophage-encoded RexAB-type phage defense system in *Pseudomonas putida*

**DOI:** 10.1038/s41598-026-36734-5

**Published:** 2026-01-21

**Authors:** Sirli Rosendahl, Anu Kängsep, Andres Ainelo, Anita Lipu, Hedvig Tamman, Rita Hõrak

**Affiliations:** Institute of Molecular and Cell Biology, University of Tartu, Tartu, Estonia

**Keywords:** Prophage, Phage defense system, RexAB, CEPEST collection, *Pseudomonas putida*, Microbiology, Molecular biology

## Abstract

Temperate bacteriophages in their prophage form are abundant residents in bacterial genomes. As the fitness of a prophage depends on the welfare of its host, prophages often encode genes that enhance their host’s fitness, thereby also contributing to their own survival. One such strategy for mutual survival is to protect the host from superinfection by other phages. We recently showed that four cryptic prophages contribute to the phage resistance of *Pseudomonas putida*, a metabolically versatile and industrially promising model bacterium. Here, we show that prophages 1, 2, and 3 exhibit anti-phage activity against specific phages in our CEPEST collection. Focusing on P1-mediated phage defense, we uncover that P1 genes PP_5643 and PP_5644 encode a two-component RexAB-type phage immunity system. The RexA_pp_ is a DNA-binding protein that shares structural similarity with phage lambda RexA. RexB_pp_ is a membrane protein that acts as a cell growth-suppressing effector when stimulated by RexA_pp_ overexpression, and probably by phage infection. The activation of RexB_pp_ results in membrane damage, as evidenced by increased membrane permeability to SYTOX Green and propidium iodide, as well as leakage of cytoplasmic enzymes. Our data suggest that RexB_pp_ causes growth cessation of infected bacteria and thus aborts the infection.

**Supplementary Information:**

The online version contains supplementary material available at 10.1038/s41598-026-36734-5.

## Introduction

Phages, both lytic and temperate, play a major role in shaping bacterial populations. Prophages, the temperate phages integrated and maintained within their bacterial host genome, are highly abundant in bacteria, being identified in up to 75% bacterial genomes^1–3^. While the impact of lytic phages on their host fitness is clearly negative, the interplay between prophage and its host is more complex. As the survival of the prophage is tightly intertwined with the host’s welfare, the relationships between temperate phages and the bacterium are mutualistic, and prophages often increase their host’s fitness^4^ by contributing to its virulence^5–7^, resistance to antibiotics^8,9^ or phages^10–14^.

Recent research on the bacteria-phage interactions has unveiled an amazing diversity of anti-phage mechanisms in bacteria^10,14–17^, but has also revealed the equally diverse arsenal of counter-defense systems in phages that enable them to evade the bacterial immunity mechanisms^18,19^. After sensing the phage infection, the bacterial immune systems can directly interfere with the phage life cycle and stop phage proliferation without affecting the host’s fitness. Such immunity systems, for instance, include the restriction-modification and CRISPR-Cas systems, which recognize and cleave the invading phage DNA^20,21^. The other type of phage defense systems, the abortive infection (Abi) systems, counter phage attacks by inhibiting the vital functions of the infected cell, leading to cell growth cessation or even cell death^22–24^. While the Abi systems harm infected cells, they efficiently prevent phage proliferation in the bacterial population. A plethora of different Abi mechanisms are described, involving growth arrest due to the degradation of cellular RNA^25,26^ or proteins^27^, phosphorylation of proteins^28^, or disruption of cell membrane integrity^29,30^.

One of the first Abi systems to be described was the RexAB two-component system encoded by the λ prophage^29^. The system, consisting of DNA-binding RexA^31,32^ and membrane-located RexB^29^, inhibits the growth of several lambdoid phages^33^. RexA is supposed to detect phage replication intermediates, and its binding to RexB triggers the opening of the RexB ion channel, resulting in the loss of membrane potential and the hydrolysis of ATP^29,34,35^. Thus, RexA acts as an infection sensor, and RexB as an effector leading to the Abi phenotype. While some studies suggest that activation of RexB leads to cell death^29^, others propose the induction of a stationary phase-like state that protects the host against phage infection^36^. RexAB-type Abi systems have been recently found in *Mycobacterium* and *Gordonia* prophages Sbash and CarolAnn, respectively^37,38^. While the Abi mechanism of the Sbash- and CarolAnn-encoded systems seems to resemble that of the λ RexAB, the mycobacteriophage Butters encodes a distinct RexAB-type Gp30/Gp31 system, where the anti-phage effector is rather a RexA-resembling cytosolic protein, not the membrane counterpart of the system^39^.

*Pseudomonas putida*, a water, soil, and rhizosphere bacterium, has long served as a model organism in molecular biology and a workhorse for industrial biotechnology^40^. The most well-examined and biotechnologically promising *P. putida* strains are KT2440 and its isogenic PaW85, which harbor four prophages in their chromosomes^41,42^. These prophages make up 2.6% of the *P. putida* genome and carry a fitness cost to the bacterium under the conditions of DNA damage, as removing all prophage regions resulted in increased tolerance of *P. putida* to UV light and other DNA insults^41^. Although many prophage genes are activated during DNA stress^43^, all four prophages seem to be cryptic, as they do not produce infectious virus particles nor cause cell lysis^41,44^.

While prophages decrease *P. putida* fitness under DNA stress conditions, our recent research has shown that they are advantageous for the bacterium under conditions of phage attack^45^. Screening the 67 *P. putida* phages in the CEPEST collection revealed that the absence of four cryptic prophages sensitized *P. putida* to many phages in our collection^45^. Here, we aimed to decipher which prophages are engaged in the anti-phage effect. We demonstrate that prophages 1, 2, and 3 exhibit anti-phage activity against specific phages in our CEPEST collection, whereas P4 is not involved in phage defense. Focusing on prophage P1-mediated phage defense, we demonstrate that P1 genes PP_5643 and PP_5644 encode a RexAB-type phage defense system.

## Results

### Three *P. putida* prophages offer phage-specific protection

**Fig. 1:**
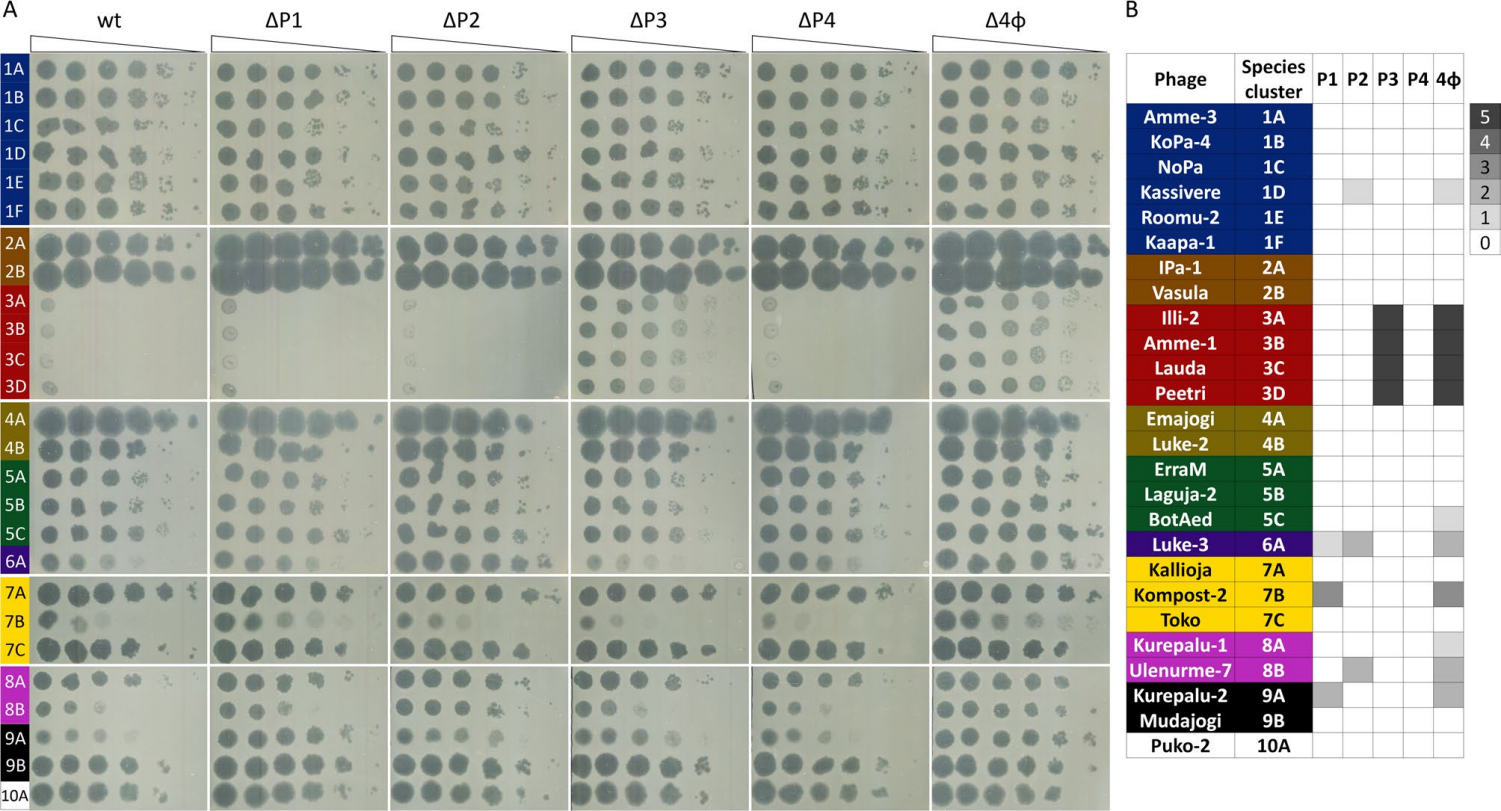
Specificity of prophage-mediated phage defense. (**A**) The efficiency of plating (EOP) of phages was determined on bacterial lawns of *P. putida* PaW85 wild-type (wt) and of its derivatives lacking prophage P1 (ΔP1), P2 (ΔP2), P3 (ΔP3), P4 (ΔP4), or all four prophages (Δ4φ). Ten-fold serial dilutions of phages were spotted in 1.5 μL volumes on bacterial lawns, and plates were incubated overnight at 20 °C. (**B**) The heatmap shows the level of protection provided by prophages. 0 indicates the same EOP as in the wild-type strain, and numbers (and increasing density of grey color) indicate a tenfold increase in EOP compared to the wild-type strain.

Four *P. putida* cryptic prophages together provide a variable level of protection against several CEPEST collection phages^45^. To determine the anti-phage effect of each prophage separately, the phage sensitivity of single prophage-deficient derivatives was analyzed and compared to *P. putida* wild-type and the Δ4φ strain, which lacks all four prophages. Screening 26 representative phages of 10 genera revealed that prophages P1, P2, and P3, but not P4, contribute to defense against the tested phages (Fig. 1). Prophage P1 provides 1000-fold protection against phage Kompost-2 from species cluster 7B and also contributes to the defense against 9A and 6A phages (Fig. 1). P2 increases resistance against phages from species clusters 1D, 6A, and 8B 10–100 times (Fig. 1). P3 provides the highest protection, up to 10^5^-fold, but only against jumbo phages from genus G3.

Comparison of the results of the prophage single deletion strains with those determined in Δ4φ revealed that in most cases, a single prophage was responsible for the observed anti-phage effect. 6A Luke-3 was the only phage where both the prophages P1 and P2 contributed to defense (Fig. 1). Interestingly, there were two phages, BotAed and Kurepalu-1, where the impact of the single prophage deletion mutant on phage infectivity could not be detected, although the Δ4φ strain was still slightly more susceptible than the wild-type *P. putida*. This suggests that multiple prophages can collectively enhance protection against these phages. Going forward, we focused our attention on prophage P1 and aimed to elucidate the mechanisms behind the observed relatively broad and potent phage defense.

### Prophage P1-encoded HicAB2 is a functional toxin-antitoxin system but is not implicated either in P1 stability or in phage defense

*P. putida* prophage P1, spanning from PP_3849 to PP_3920, is about 55 kb long and contains 78 ORFs, according to the *Pseudomonas* Genome Database^46^. Interestingly, P1 contains a toxin-antitoxin operon, *hicAB2* (PP_3900-3899), and when we tried to delete this TA system, the whole P1 prophage was excised from the *P. putida* genome^45^. This indicated that the HicAB2 TA system may be involved in the stable maintenance of P1 in the chromosome. Given that TA systems can act as Abi systems to inhibit phage propagation in bacterial populations^23^, we decided to test the potential role of the HicAB2 locus in both P1 stability and phage defense.

**Fig. 2:**
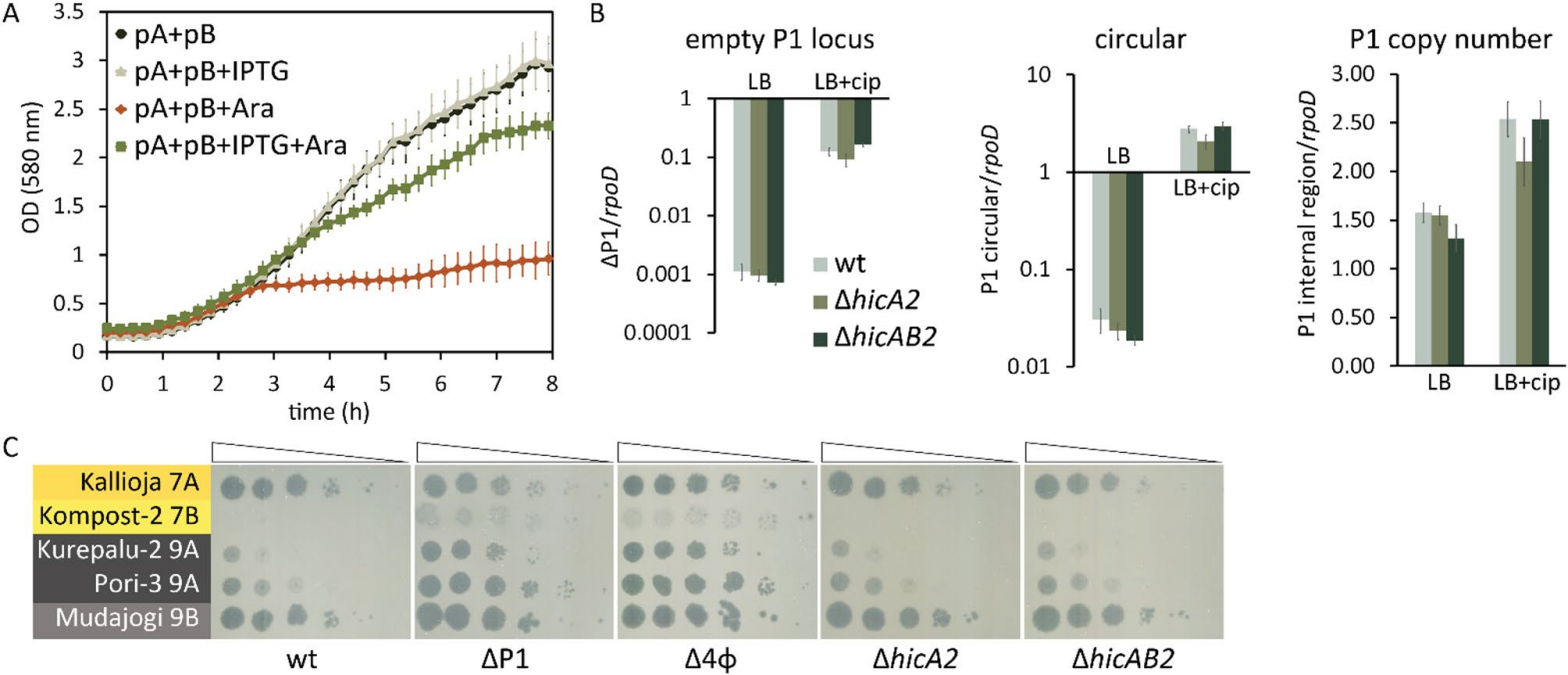
HicAB2 is a functional TA system but is not involved in the P1 stability or the phage resistance of *P. putida*. (**A**) The *hicAB2* locus encodes a functional TA module. Growth curves of *E. coli* DH5α cells harboring the pBRlacItac-hicB2 (pB) and pBAD33-hicA2 (pA) plasmids for antitoxin HicB2 and toxin HicA2 overexpression, respectively. Antitoxin production was induced with 5 mM IPTG (+ IPTG), and toxin production with 10 mM arabinose (+ Ara). The average of six biological replicates, along with the standard deviation, is presented. (**B**) The *hicAB2* locus is not involved in P1 stability. The excision frequency of P1, relative abundance of the extrachromosomal P1 circle, and the P1 genome in *P. putida* wild-type (wt), ∆*hicA2,* and ∆*hicAB2* strains were determined with qPCR. *rpoD* was used as the reference gene. Bacteria were grown in LB or LB supplemented with 0.03 µg/mL ciprofloxacin (+ cip) for ~ 18 h, after which genomic DNA was extracted for qPCR assay. The results are the average of at least five biological replicates with a 95% confidence interval. There was no statistically significant difference between wild-type and the deletion strains under any conditions (p = 0.001, Student’s T-test). (**C**) The *hicAB2* locus is not engaged in anti-phage defense. The plating efficiency of G7 and G9 genus phages was determined on bacterial lawns of *P. putida* wild-type PaW85 (wt), ∆P1, ∆4φ, ∆*hicA2*, and ∆*hicAB2* strains.

First, we tested whether the *hicA2* and *hicB2* constitute a functional toxin-antitoxin system by ectopically expressing the *hicAB2* genes in *E. coli*. For that, the *hicB2* antitoxin gene was cloned under the control of IPTG-inducible *tac* promoter in plasmid pBRlacItac, and the *hicA2* toxin under the control of arabinose-inducible pBAD promoter in plasmid pBAD33. The cloning of *hicA2* into pBAD33 was possible only if *E. coli* competent cells contained the plasmid for expression of HicB2 antitoxin, indicating that *hicA2* encodes a toxic protein. In line with that, induction of *hicA2* expression with arabinose resulted in growth suppression (Fig. 2A). Concurrent induction of toxin and antitoxin alleviated the HicA2-caused growth inhibition, confirming that HicB2 counteracted the toxic effects of HicA2 (Fig. 2A). Expression of antitoxin alone did not affect bacterial growth. These data confirm that the *hicAB2* locus encodes a functional TA module.

To test the effect of the HicAB2 system on P1 stability and phage resistance in *P. putida*, we attempted to delete the entire TA operon or the toxin gene *hicA2* from the P1 genome. Similar to our previous attempts to delete the *hicAB2* operon^45,47^, the P1 prophage was easily lost from the *P. putida* chromosome. Still, we could eventually pick up some toxin-deficient Δ*hicA2* clones with P1 maintained in its chromosomal location by analyzing several dozen recombinants. Only about 10% of recombinants turned out to be Δ*hicA2* strains, while in most clones, P1 had been excised from the *P. putida* genome. The obtained Δ*hicA2* strain was used to create the Δ*hicAB2* deletion strain. Again, for capturing the Δ*hicAB2* strain, we needed to analyze many recombinants, as in about 90% of clones, the P1 had been excised from its chromosomal locus. Given that deletion of *hicAB2* seemed to provoke P1 excision, we hypothesized that the HicAB2 system might be involved in the stable maintenance of P1 in the *P. putida* genome. To test this, we compared the stability of P1 in *P. putida* wild-type, Δ*hicA2,* and Δ*hicAB2* strains using qPCR, with *rpoD* as a reference gene. Considering that many P1 genes are activated during DNA damage^43^, we examined P1 excision in non-stressed bacteria and bacteria subjected to ciprofloxacin-induced DNA stress. Probing for loss of P1 with primers flanking the prophage genome showed that the spontaneous excision of P1 occurs at a frequency of about 10^–3^, while the ciprofloxacin treatment increased the excision rate by about two orders of magnitude (Fig. 2B). However, contrary to our expectations, the deficiency of the TA system did not influence either the spontaneous or the DNA-damage-induced excision of P1 (Fig. 2B). To test whether the excised P1 can form an extrachromosomal circle or is lost from the cells, qPCR analysis of the junction between the two ends of the predicted prophage circle and a DNA region inside the prophage was performed. The extrachromosomal P1 circle was detected in non-stressed wild-type, Δ*hicA2,* and Δ*hicAB2* strains at a similar frequency of about 2 × 10^–2^, i.e., in 2% of bacteria, which is about 25-fold higher than the spontaneous excision frequency of P1 (Fig. 2B). If bacteria were treated with ciprofloxacin, then the copy number of the prophage circle increased about 100-fold in all tested strains (Fig. 2B). These data indicate that the excised P1 can efficiently exist as an extrachromosomal element and that the HicAB2 system is not involved in P1 stability. Corroborating this result, qPCR analysis of a DNA region inside the prophage verified that the prophage copy number was not influenced by the absence of the *hicAB2* locus (Fig. 2B). Interestingly, the copy number of P1 was higher than that of the reference gene *rpoD*, suggesting that cells can simultaneously contain both the chromosomal and extrachromosomal copies of P1 prophage.

Next, we investigated whether the HicAB2 system was responsible for the P1-mediated defense against 7B and 9A phages. As the efficiency of plaquing of 7B and 9A phages on bacterial lawns of Δ*hicA2* and Δ*hicAB2* strains was similar to that of the wild-type, the HicAB2 system is not responsible for the P1-provided anti-phage effect (Fig. 2C). Thus, while the HicAB2 is a potent TA system, it does not contribute to P1 stability or the phage defense provided by P1.

### PP_5643-5644 operon codes for phage defense genes

**Fig. 3:**
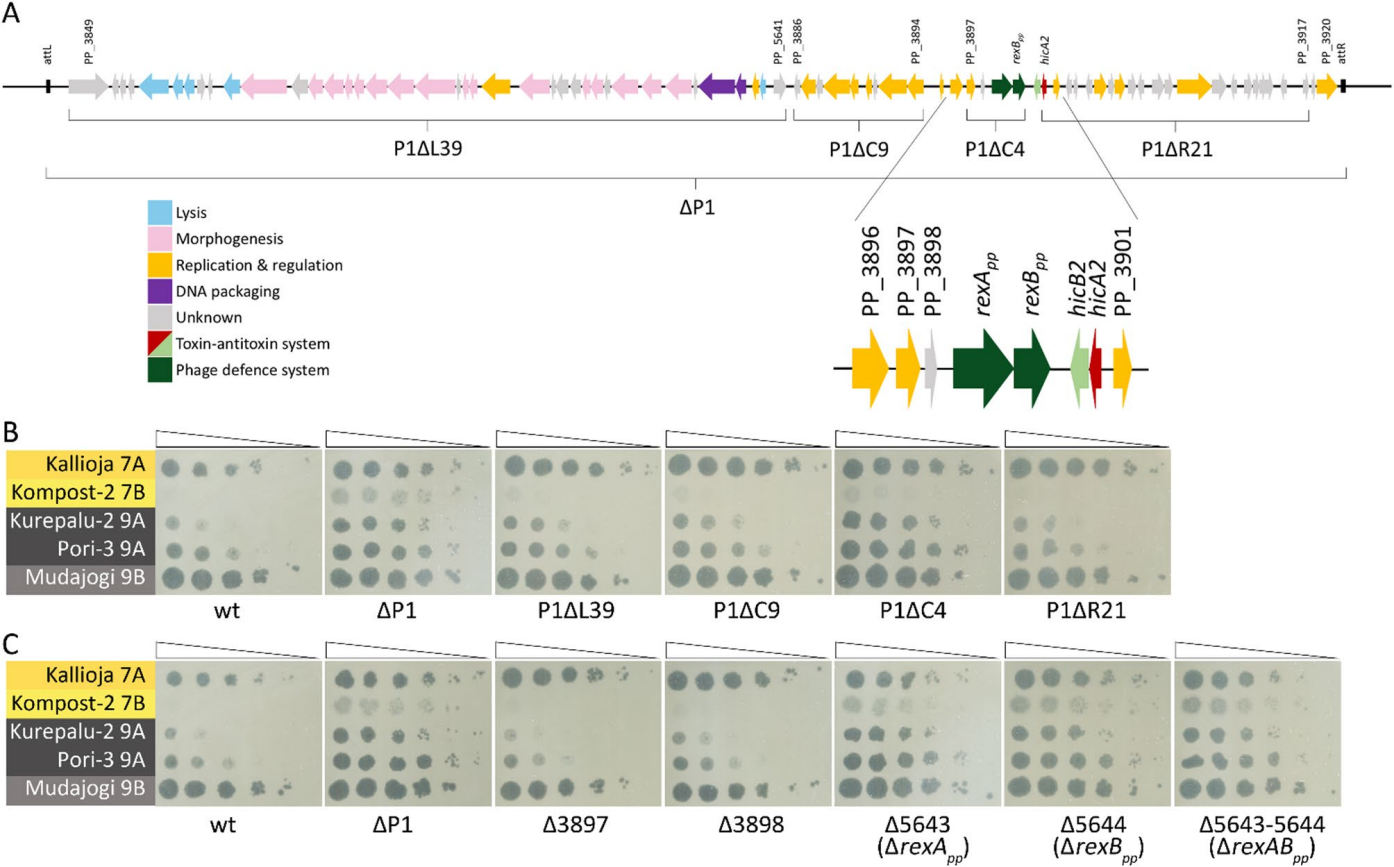
PP_5643 and PP_5644 constitute an anti-phage system. (**A**) Schematic representation of prophage P1. Genes deleted from various deletion derivatives are indicated with brackets. (**B**) The plating efficiency of G7 and G9 genus phages on *P. putida* wild-type (wt) and its ∆P1, P1∆L39, P1∆C9, P1∆C4, and P1∆R21 derivative strains. (**C**) The plating efficiency of G7 and G9 genus phages on *P. putida* wild-type (wt) and its deletion derivative strains ∆P1, ∆3897, ∆3898, ∆5643 (*∆rexA*_*pp*_), ∆5644 (*ΔrexB*_*pp*_), ∆5643–5644 (*ΔrexAB*_*pp*_).

To identify the P1-encoded phage defense locus, we constructed several deletion strains with different regions of the prophage P1 genome missing (Fig. 3A). Notably, as with deletions of the *hicAB2* locus, deletions in other prophage regions also induced P1 excision, with typically 50–70% of recombinants having lost the entire P1. The four deletion derivative strains, P1ΔL39, P1ΔR21, P1ΔC9, and P1ΔC4, lacked different numbers of P1 genes—39, 21, 9, and 4 genes, respectively (Fig. 3A). Phage infection tests revealed that the strains P1ΔL39, P1ΔR21, and P1ΔC9 retained the anti-phage defense similar to the wild-type (Fig. 3B). However, the P1ΔC4 strain behaved similarly to the ΔP1 strain, demonstrating higher susceptibility against the infection of 7B and 9A phages (Fig. 3B). The strain P1ΔC4 lacks four genes: PP_3897, PP_3898, PP_5643, and PP_5644, which are located just downstream of a putative Cro/CI family transcriptional repressor gene PP_3896 (Fig. 3A). We created the single deletion strains and the Δ5643-5644 double deletion strain to define which genes could be responsible for the anti-phage defense. Phage infection analyses demonstrated that deleting PP_5643, PP_5644, or both sensitized *P. putida* to phage infection, while deleting either PP_3897 or PP_3898 did not (Fig. 3C). This indicates that for the anti-phage effect against 7B and 9A phages, both PP_5643 and PP_5644 are required.

### PP_5643-5644 system shares similarities with the phage λ RexAB phage exclusion system

**Fig. 4:**
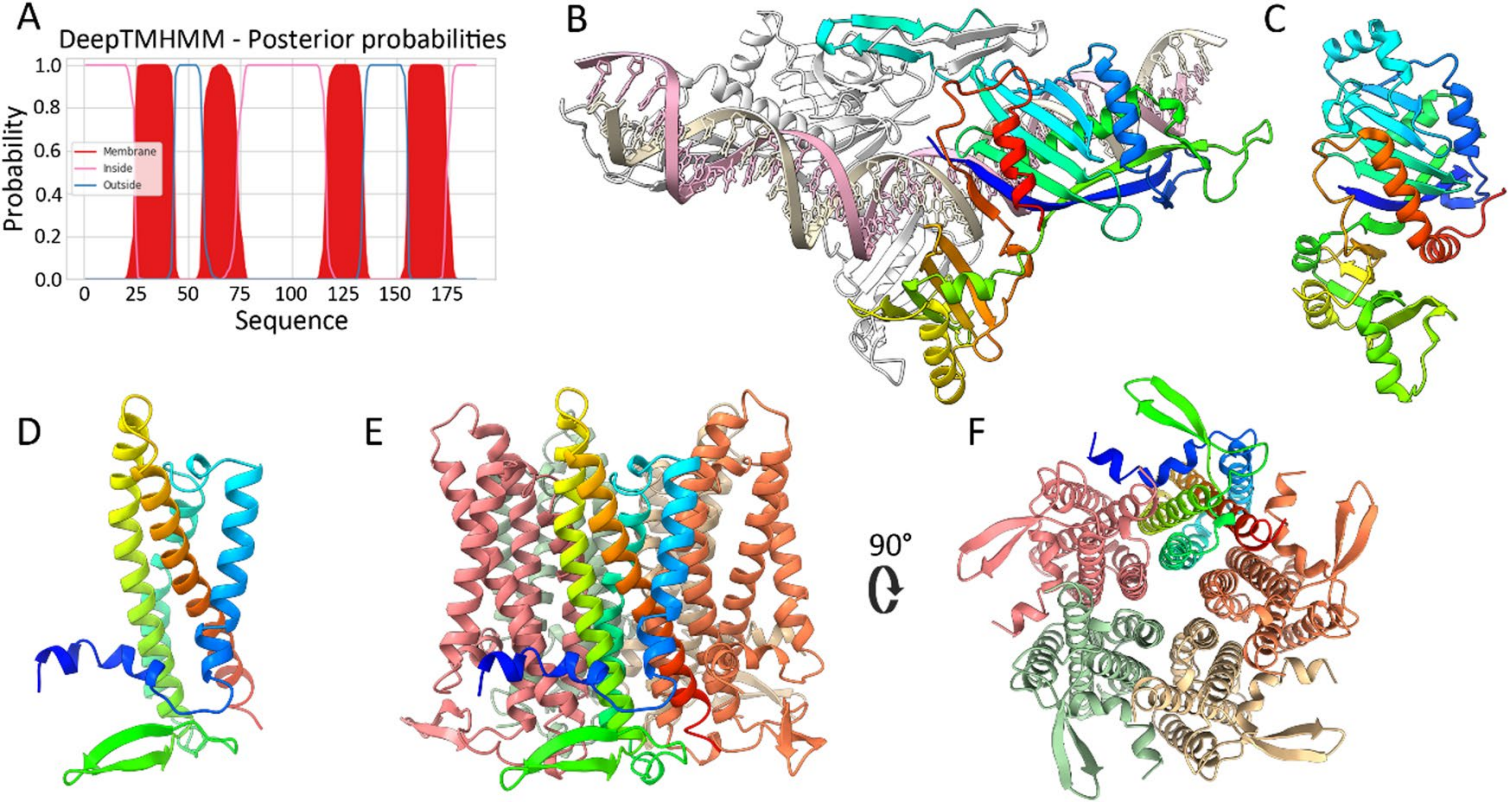
Bioinformatic characterization of PP_5643 and PP_5644. (**A**) DeepTMHMM prediction for transmembrane helices of PP_5644. (**B**) AlphaFold 3 model of a PP_5643 dimer:dsDNA complex. One monomer of PP_5643 is rainbow-colored (blue to red from the N to C terminus), and the 2^nd^ monomer is white. (**C**) Crystal structure of phage λ RexA (PDB 8TWQ^32^), aligned with PP_5643 in DALI and colored analogously. (**D**) AlphaFold 3 model of PP_5644 monomer. (**E**) AlphaFold 3 model of PP_5644 pentamer. Each monomer is presented with a different color, and one monomer is rainbow-colored. (**F**) AlphaFold 3 model of PP_5644 pentamer, viewed from the end predicted to be cytoplasmic by DeepTMHMM.

We performed various bioinformatic analyses to gain insight into the structural and functional features of PP_5643 and PP_5644. PP_5643 is annotated as cytoplasmic, and PP_5644 as an inner membrane protein^46^. Analysis of PP_5644 with DeepTMHMM^48^ predicted that the protein has four putative transmembrane domains (Fig. 4A). BLAST analysis did not yield any insights into the functionalities of PP_5643 and PP_5644, as the main identified hits for both were hypothetical proteins of different pseudomonads. Interestingly, however, protein homology search with HHpred revealed the lambda phage RexA protein as a highly probable homologue for PP_5643 (probability 98.19; E-value 3.2e-5). The finding that PP_5643 could be related to RexA is highly reasonable in the context of phage defense because RexA and RexB constitute a well-known two-component phage exclusion Abi system that prevents bacteriophage T4 *rII* mutants from growing in *E. coli* λ phage lysogens^29^. Although HHpred did not detect any probable homologues to PP_5644, a functional similarity to RexB is possible, as both are membrane proteins. This suggests that PP_5643 and PP_5644 constitute a RexAB-type phage defense system. AlphaFold modeling of PP_5643 further supported its similarity to RexA, a non-specific DNA-binding protein^31^. Building models with various stoichiometries yielded the highest confidence predictions for a PP_5643 dimer:dsDNA complex (ipTM = 0.81–0.86, pTM = 0.85–0.88, depending on random 40-mer DNA sequence used). In these models, PP_5643 dimerizes as a clamp around the DNA (Fig. 4B). If the predicted PP_5643 dimer complex with DNA was used as input in the Dali structural homology search, again, RexA was identified as the most homologous structural relative (Fig. 4C, PDB: 8TWQ, Z-score 8.8, RMSD 4.2 Å).

The structural model of PP_5644 agrees with the DeepTMHMM prediction, showing a 4-helix bundle with both termini at the same end of the molecule (Fig. 4E). Considering the possibility that PP_5644 forms pores in the membrane, we modeled homomultimers with 2–8 protein copies. The best confidence metrics were obtained for a pentameric arrangement (Fig. 4D, ipTM = 0.74, pTM = 0.76). Hexa- and heptamer configurations yielded models that were essentially analogous expansions of the pentameric ring with respective ipTM scores of 0.69 and 0.65, which leaves the true stoichiometry uncertain and possibly variable.

The gene neighborhood of the PP_5643-5644 also resembles that of the λ phage RexAB system. The *rexAB* genes are located just downstream of λ CI repressor, and PP_5643-5644 are also found downstream of the P1 CI-like repressor PP_3896, though separated from that by two additional genes (Fig. 3A). Given these similarities, we suggest that PP_5643-5644 is a RexAB-type phage defense system and will refer to it as RexAB_pp_.

### Two-hybrid analysis of RexA_pp_ and RexB_pp_ interactions

**Fig. 5:**
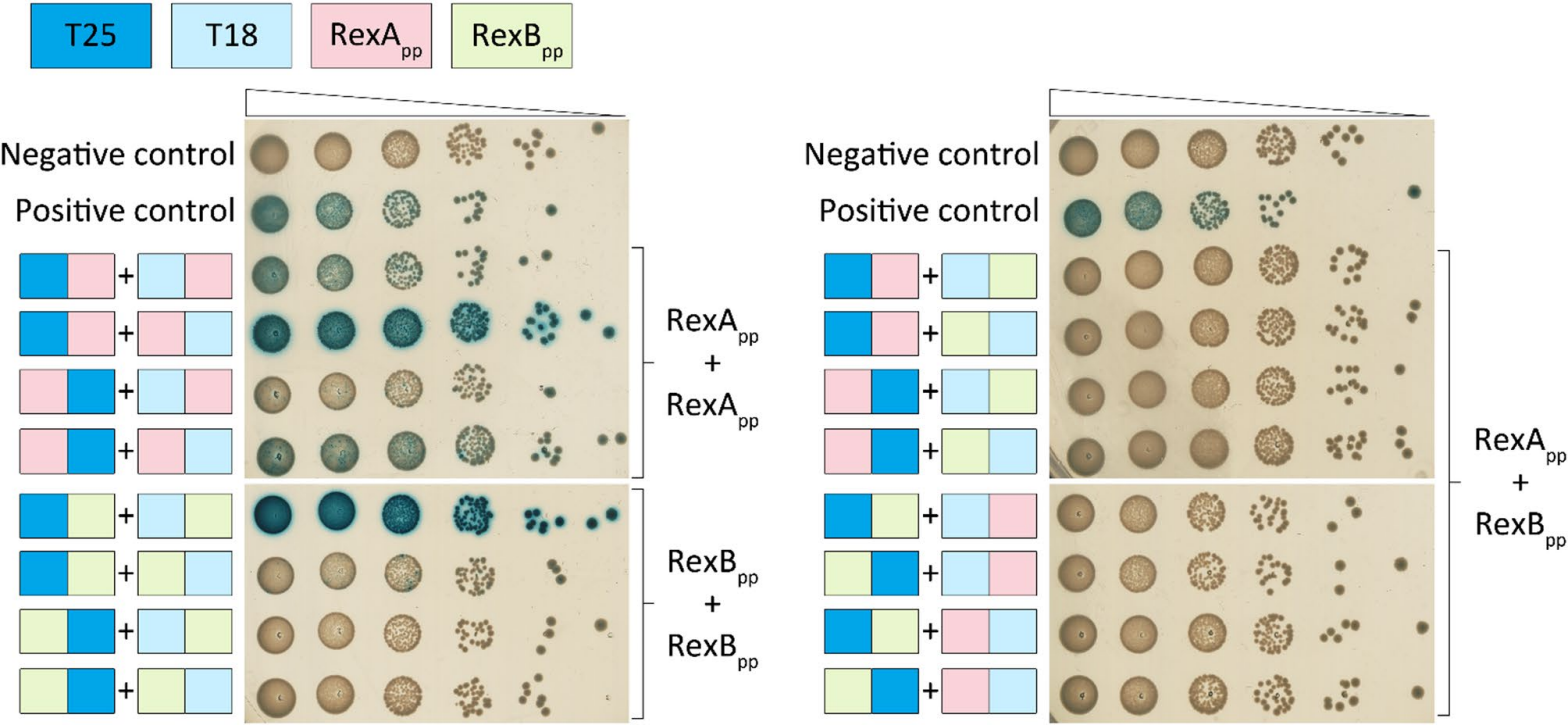
RexA_pp_ and RexB_pp_ interactions were detected using the two-hybrid analysis*.* The T25 fragment (aa 1–224, dark blue) of *Bordetella pertussis* adenylate cyclase *cyaA* in plasmid pT25 or the T18 fragment (aa 225–399, light blue) in plasmid pT18 was fused to RexA_pp_ (pink) or RexB_pp_ (green) at either the N- or C-terminus. Pairwise combinations of the plasmids were co-transformed into *E. coli cyaA*-defective tester strain BTH101 and tenfold dilutions of bacteria were spotted on selective media containing ampicillin, kanamycin, X-gal, and IPTG. Negative control contains pT25 and pT18 plasmids without fused proteins, and positive control contains plasmids pT25-graT and pT18-graA, which express *cyaA* fusions with the toxin GraT and the antitoxin GraA^50^. The plates were incubated for 42 h at 30 °C. If the proteins interact, the colonies turn blue.

The anti-phage activity of λ RexAB has been proposed to be triggered after RexA detects phage DNA intermediates, leading to RexA binding and activating the membrane protein RexB^29,35^. Two-hybrid analysis has demonstrated that lambda RexA and RexB indeed interact^35^. AlphaFold predictions suggest that the P1-encoded RexA_pp_ can form a dimer, and RexB_pp_ a multimer (Fig. 4). To test this experimentally and to assay the possible interactions between the RexA_pp_ and the RexB_pp_, we used an in vivo BACTH assay, which relies on the activation of the *lacZ* reporter in an adenylate cyclase *cyaA*-deficient strain^49^. For that, the RexA_pp_ and RexB_pp_ were fused to the N- or C-terminus of both the *cyaA* T18 and T25 domains. Altogether, eight plasmids were constructed, and pairwise combinations of plasmids were tested in a *cyaA*-defective *E. coli* strain BTH101 (Fig. 5). Co-expression of different CyaA-RexA_pp_ fusions verified that RexA_pp_ can dimerize, as three out of the four possible fusion protein combinations were positive in β-galactosidase assay (Fig. 5). RexB_pp_ also interacts with itself, although only one combination of fusion proteins demonstrated strong interaction in the β-galactosidase assay (Fig. 5). Namely, the interaction between RexB_pp_ could be detected only when the T18 or T25 fragments were joined to the N-terminus of RexB_pp_, meaning that a free C-terminus is necessary for RexB_pp_ oligomerization. To test whether the RexA_pp_ and the RexB_pp_ can interact with each other, the eight possible RexA_pp_ and RexB_pp_ plasmids were tested pairwise. None of these combinations could activate the *lacZ* reporter in the tester strain (Fig. 5), indicating that RexA_pp_ and RexB_pp_ do not interact, at least when fused with *cyaA* fragments. Still, two-hybrid analysis verified the AlphaFold prediction that both the RexA_pp_ and the RexB_pp_ can dimerize or form higher-order oligomers.

### RexA_pp_ is a DNA-binding protein

**Fig. 6:**
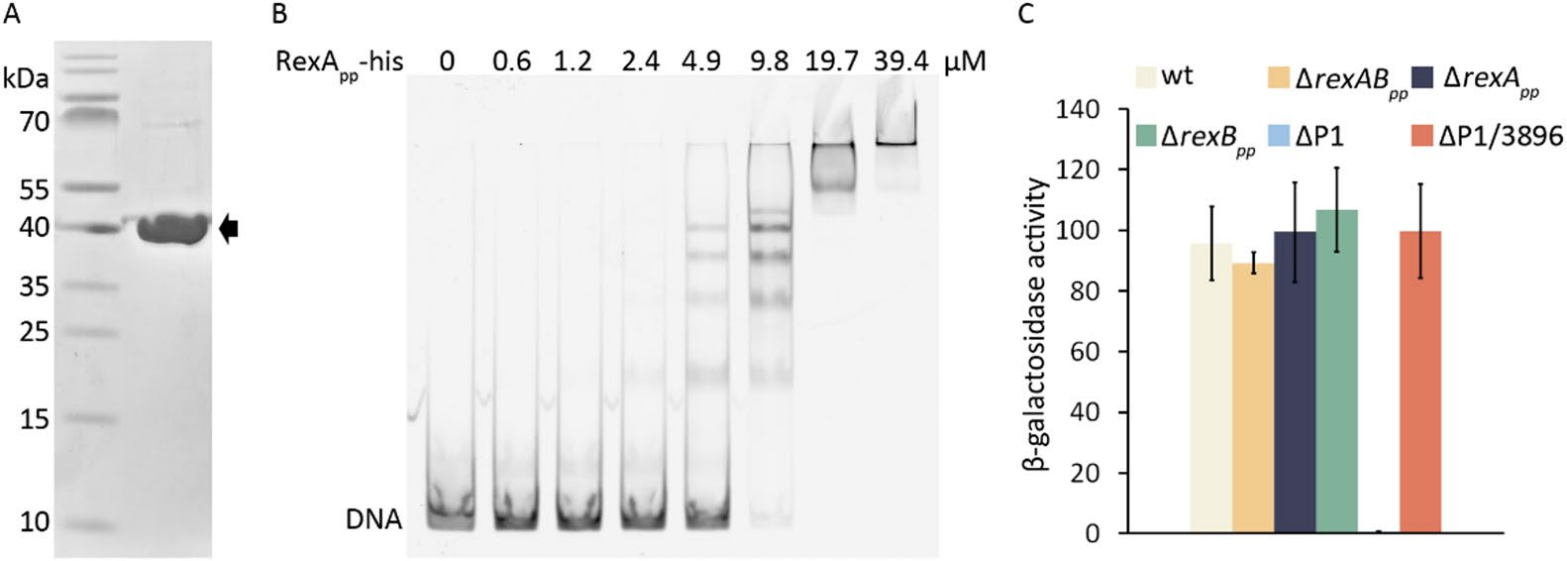
RexA_pp_ is a DNA-binding protein. (**A**) Purified RexA_pp_-His (Mw 37.5 kDa) protein. (**B**) EMSA analysis of RexA_pp_-His. The indicated concentration of RexA_pp_-His was mixed with 0.6 µM FAM-labeled DNA fragment 1 (175 bp). Following incubation at room temperature for 30 min, the reaction mixtures were resolved on 5% native polyacrylamide gel. (**C**) Transcriptional fusion of the PP_3896 to the *lacZ* gene in a plasmid p9TT_B_3896-lacZ was analyzed in *P. putida* wild-type (wt), Δ*rexA*_*pp*_, Δ*rexB*_*pp,*_ Δ*rexAB*_*pp*_, ΔP1, and ΔP1/3896 strains. The results represent the average of five biological replicates with standard deviation. The gel images are cropped to show the relevant lanes. The original gels are presented in Supplementary Figure S2.

To test whether RexA_pp_ can bind DNA as suggested by the AlphaFold prediction (Fig. 4B), a His_6_-tag was fused to the C-terminus of the protein, and the RexA_pp_-His (Mw 37.5 kDa) was overexpressed and purified (Fig. 6A). EMSA analyses showed that RexA_pp_ can bind to different DNA fragments (Fig. 6B, EMSA with one DNA probe is presented), showing that, like λ RexA, the *P. putida* P1-encoded RexA_pp_ is a non-specific DNA-binding protein. Under high protein-DNA ratios, several supershifted bands were revealed in the EMSA gels, indicating that several RexA_pp_ molecules (most probably protein dimers) bind to one DNA molecule (Fig. 6B). This is not surprising, given that the EMSA DNA fragment is 175 bp long. According to the model (Fig. 4B), one RexA_pp_ dimer covers approximately 40 base pairs in total and the distance between two dimers could be as low as 25 bp, if the neighboring dimers would overlap on opposite sides of DNA.

Lambda RexA has been shown to modulate the bistable switch between phage lysogeny and lytic pathways, which involves RexA binding and repressing the *P*_RM_ promoter of the CI repressor^31,32^. To test whether *P. putida* RexA_pp_ can influence the expression of PP_3896, the putative repressor gene of P1 and the first gene in the operon that also contains *rexAB*_*pp*_ (Fig. 3A), the PP_3896-*lacZ* transcriptional fusion was constructed and introduced into *P. putida* wild-type, Δ*rexA*_*pp*_, Δ*rexB*_*pp,*_ Δ*rexAB*_*pp*_, and ΔP1 deletion strains. Enzyme measurements demonstrated equally high β-galactosidase activities in *P. putida* wild-type and Δ*rexAB*_*pp*_ deletion strains, while the PP_3896 promoter activity was almost undetectable in the P1 deletion strain (Fig. 6C). This shows that PP_3896 expression is not influenced by *rexAB*_*pp*_ and that a P1-encoded factor is necessary for the transcriptional activation of PP_3896. Drawing parallels with lambda regulation, this factor is probably PP_3896 itself. To test this, we attempted to delete PP_3896 from the prophage P1 genome. However, despite several efforts, this was not possible, as the entire prophage P1 was always excised. Thus, we cloned PP_3896 with its native promoter region into miniTn7 and introduced the miniTn7-3896 cassette into the ΔP1 deletion strain. Analysis of PP_3896-*lacZ* transcriptional fusion demonstrated that the complementation of the ΔP1 strain with PP_3896 restores the PP_3896 promoter activity to wild-type level (Fig. 6C). Thus, similar to the lambda CI, the PP_3896 expression is also positively autoregulated.

### Overexpression of RexA_pp_ leads to RexB_pp_-dependent growth inhibition and membrane defects

**Fig. 7:**
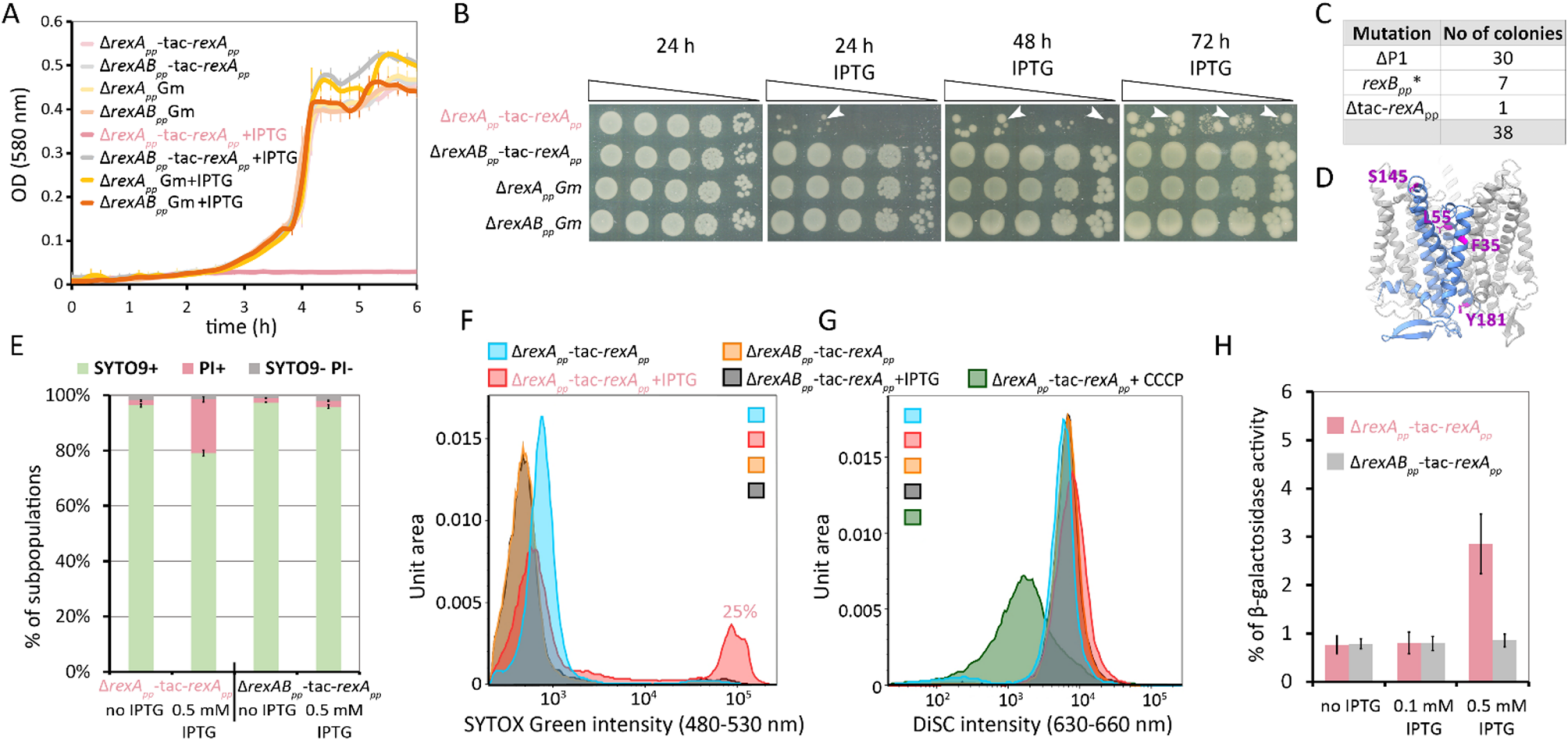
RexB_pp_ is a toxic effector activated by the overexpression of RexA_pp_. (**A**) Growth curves of *P. putida* Δ*rexA*_*pp*_-tac-*rexA*_*pp*_, Δ*rexAB*_*pp*_-tac-*rexA*_*pp*_, Δ*rexA*_*pp*_Gm, and Δ*rexAB*_*pp*_Gm strains in LB medium supplemented with gentamycin. RexA_pp_ production was induced from the tac-*rexA*_*pp*_ cassette with 0.5 mM IPTG (+ IPTG). The average of six technical replicates, along with the standard deviation, is presented. (**B**) tenfold dilutions of Δ*rexA*_*pp*_-tac-*rexA*_*pp*_, Δ*rexAB*_*pp*_-tac-*rexA*_*pp*_, Δ*rexA*_*pp*_Gm and Δ*rexAB*_*pp*_Gm on LB plates supplemented with gentamycin, with and without 0.5 mM IPTG. Images were taken 24, 48, and 72 h after incubation. White arrows indicate well-growing mutants. (**C**) Table describing different mutations found in Δ*rexA*_*pp*_-tac-*rexA*_*pp*_ mutants that appeared on LB plates supplemented with gentamycin and 0.5 mM IPTG after 72 h of growth. (**D**) Amino acid substitution mutations are shown on the AlphaFold 3 model of RexB_pp_ pentamer. (**E**) Flow cytometry analysis of SYTO 9 and PI-stained Δ*rexA*_*pp*_-tac-*rexA*_*pp*_ and Δ*rexAB*_*pp*_-tac-*rexA*_*pp*_ strains grown in liquid medium with and without 0.5 mM IPTG. Relative proportion of the subpopulations stained with SYTO 9 (SYTO9 +), propidium iodide (PI +), and non-stained (SYTO9- PI-) cells (means with 95% confidence intervals) from at least three independent determinations are presented. (**F**) Flow cytometry histograms of SYTOX Green-stained Δ*rexA*_*pp*_-tac-*rexA*_*pp*_ and Δ*rexAB*_*pp*_-tac-*rexA*_*pp*_ strains grown in liquid medium with and without 0.5 mM IPTG. Percentage of SYTOX Green-positive cells for the Δ*rexA*_*pp*_-tac-*rexA*_*pp*_ grown with 0.5 mM IPTG is indicated above the histogram. One representative result of six independent experiments is presented. (**G**) Flow cytometry histograms of DiSC_3_(5)-stained Δ*rexA*_*pp*_-tac-*rexA*_*pp*_ and Δ*rexAB*_*pp*_-tac-*rexA*_*pp*_ strains grown in liquid medium with and without 0.5 mM IPTG. To induce membrane depolarization, 10 mM CCCP (carbonyl cyanide m-chlorophenyl hydrasone) was added to the Δ*rexA*_*pp*_-tac-*rexA*_*pp*_ sample (+ CCCP). One representative result of five independent experiments is presented. (**H**) β-Galactosidase activities measured from the supernatants of the Δ*rexA*_*pp*_-tac-*rexA*_*pp*_ and Δ*rexAB*_*pp*_-tac-*rexA*_*pp*_ strains grown in liquid medium with and without 0.5 mM IPTG. The strains carry the p9TT_B_lacZ/amrZ plasmid. Data (means and standard deviation) of at least four independent measurements are presented.

To test whether the complementation of Δ5643 (Δ*rexA*_*pp*_) and Δ5644 (Δ*rexB*_*pp*_) strains with *rexA*_*pp*_ or *rexB*_*pp*_ genes can restore phage resistance, we aimed to construct minitransposons with the *lacI*^q^*-P*_*tac*_*-rexA*_*pp*_ and the *lacI*^q^*-P*_*tac*_*-rexAB*_*pp*_ expression cassettes to introduce them into *P. putida* deletion strains. While we easily obtained the plasmid with miniTn7-*lacI*^q^*-P*_*tac*_*-rexA*_*pp*_, the construction of miniTn7-*lacI*^q^*-P*_*tac*_*-rexAB*_*pp*_ was unsuccessful, indicating that even without IPTG-induction, the leaky expression of both genes was toxic to *E. coli.* When the miniTn7-*lacI*^q^*-P*_*tac*_*-rexA*_*pp*_ cassette was inserted into the *glmS* locus of the *P. putida* Δ*rexA*_*pp*_ and Δ*rexAB*_*pp*_ derivatives, we observed that the overexpression of RexA_pp_ inhibits the growth of bacteria, but only of the Δ*rexA*_*pp*_ strain and not in the Δ*rexAB*_*pp*_ genetic background (Fig. 7A, B). While the Δ*rexA*_*pp*_ and Δ*rexAB*_*pp*_ strains differ only in the presence of the *rexB*_*pp*_ gene, this suggests that not RexA_pp_ itself, but rather RexB_pp_ could be the culprit in growth inhibition. Interestingly, well-growing mutants quickly emerged from the Δ*rexA*_*pp*_-tac-*rexA*_*pp*_ strain (Fig. 7B). PCR and sequencing analysis of 38 of those mutants revealed that 30 mutants had lost the entire prophage P1 from the genome, one mutant had lost the tac-*rexA*_*pp*_ cassette, and seven mutants had a mutated *rexB*_*pp*_ gene (Fig. 7C). In two mutants, the *rexB*_*pp*_ gene was disrupted by premature stop codons (W25stop, G153stop). In one mutant, 10 nucleotides were inserted into the T103 codon, and in four mutants, an amino acid substitution had occurred (F35L, L55P, S145N, Y181H; Fig. 7D). Thus, to relieve the growth defect caused by RexA_pp_ overexpression, the *rexB*_*pp*_ gene in the chromosome should be deleted or mutated, or RexA_pp_ expression should be interrupted. This suggests that the growth-suppressing effector is the membrane protein RexB_pp_, and that overexpression of RexA_pp_ triggers its activation.

Although the overexpression of RexA_pp_ with 0.5 mM IPTG suppressed the growth of Δ*rexA*_*pp*_ cells, the bacteria’s growth was unaffected without induction (Fig. 7A, B) and with low IPTG concentrations (Supplementary Figure S1A). Thus, we tried to test whether the *trans*-encoded RexA_pp_ can complement the phage resistance of the Δ*rexA*_*pp*_ strain under conditions where bacterial growth is not suppressed. Induction of RexA_pp_ with 0.01 mM IPTG did not affect the growth of the Δ*rexA*_*pp*_-tac-*rexA*_*pp*_ strain at 20 °C (Supplementary Figure S1A). Therefore, we used this IPTG concentration for inducing RexA_pp_ in the infection experiments with 9A Kurepalu-2 phage. The data obtained revealed no restoration of phage defense in the Δ*rexA*_*pp*_ strain (Supplementary Figure S1B). On the contrary, the Δ*rexA*_*pp*_-tac-*rexA*_*pp*_ strain was even more sensitive to Kurepalu-2 infection than the Δ*rexA*_*pp*_ strain (Supplementary FigureS1B). Thus, if RexA_pp_ is expressed in *trans*, then it cannot complement the phage defense defect of the Δ*rexA*_*pp*_ strain.

Given that RexB_pp_ is a membrane protein and drawing parallels with λ RexB, we hypothesize that its activation may result in membrane defects. To investigate this possibility, we tested RexA_pp_-overexpressing *P. putida* strains for membrane integrity by analyzing bacteria stained with SYTO 9 and propidium iodide (PI) using flow cytometry. PI cannot usually cross intact membranes, meaning that its entry into bacterial cells indicates membrane injury. The single-cell analysis revealed that RexA_pp_ overexpression in the Δ*rexA*_*pp*_ strain resulted in the emergence of a subpopulation of PI-permeable cells (Fig. 7E). As the RexA_pp_ overexpression did not change the PI-staining of the Δ*rexAB*_*pp*_-tac-*rexA*_*pp*_ strain (Fig. 7E), the RexB_pp_ activation by high levels of RexA_pp_ should be the cause of the increased PI-permeability of the Δ*rexA*_*pp*_-tac-*rexA*_*pp*_ strain. We also stained bacteria with SYTOX Green, which only enters bacteria with leaky membranes^51^. Flow cytometry analysis of SYTOX Green-stained bacteria also revealed that RexA_pp_ overexpression causes RexB_pp_-dependent membrane damage, as a subpopulation of highly fluorescent cells was detected in the IPTG-induced Δ*rexA*_*pp*_-tac-*rexA*_*pp*_ strain (Fig. 7F). To test whether the membrane damage exemplified by the increased entrance of PI and SYTOX Green also leads to membrane depolarization, the bacteria were stained with membrane polarization-sensitive dye DiSC_3_(5)^52^, but no difference between the Δ*rexA*_*pp*_-tac-*rexA*_*pp*_ and Δ*rexAB*_*pp*_-tac-*rexA*_*pp*_ strains was detected (Fig. 7G). This suggests that activated RexB_pp_ is not causing membrane depolarization.

We next asked whether these RexA_pp_-triggered and RexB_pp_-caused membrane defects can lead to cell death. Cell lysis can be measured by determining the leakage of cytoplasmic enzymes, such as the easily measurable β-galactosidase, into the supernatant of the liquid culture. Thus, we introduced a β-galactosidase expression plasmid into the *P. putida* Δ*rexA*_*pp*_-tac-*rexA*_*pp*_ and Δ*rexAB*_*pp*_-tac-*rexA*_*pp*_ strains and measured the β-galactosidase activity from the cell-free supernatants of the exponential phase bacteria. Without IPTG induction and with 0.1 mM IPTG, approximately 1% of the total β-galactosidase activity was detected in the supernatants of both strains (Fig. 7H). However, about threefold higher β-galactosidase activity was recorded in supernatants of the Δ*rexA*_*pp*_-tac-*rexA*_*pp*_ strain under the condition of 0.5 mM IPTG induction (Fig. 7H). This suggests that at least some RexB_pp_-containing cells lyse when RexA_pp_ is overexpressed. Still, the leakage of β-galactosidase from the cells is small, indicating that RexB_pp_ activation does not usually lead to cell lysis. The data in Fig. 7B also suggest that RexA_pp_-triggered activation of RexB_pp_ does not inevitably lead to cell death, but rather a severe growth suppression, as evidenced by the emergence of slowly growing colonies on the plate after 72 h (Fig. 7B).

Together, these data indicate that in the RexAB_pp_ system, RexB_pp_ is an effector protein that can compromise the membrane integrity, resulting in growth cessation. RexB_pp_ is activated by RexA_pp_ overexpression and probably also under conditions of phage infection.

## Discussion

Prophages often encode genes that restrict the superinfection of related phages and carry immunity genes against unrelated phages^10^. Our recent study demonstrated that the deletion of four cryptic prophages from the *P. putida* PaW85 genome renders the bacterium more sensitive to several phages from the CEPEST collection^45^. Here, we screened the single prophage deletion strains and demonstrated that three prophages out of four, P1, P2, and P3, are involved in phage defense. We focused on deciphering the prophage P1-provided defense mechanism and identified a two-gene locus as the effector that protects against three different CEPEST collection phage species. As the locus shares several similarities with the phage lambda RexAB phage exclusion system, we named it RexAB_pp_.

The lambda RexAB system was the first Abi system associated with anti-phage defense, and its discovery dates back already 70 years, when Seymour Benzer observed that T4 *rII* mutants cannot infect *E. coli* K12 λ lysogens^53^. However, despite the numerous studies conducted, the exact mechanism by which the RexAB system is activated by the T4 *rII* mutant phage remains unknown. Still, the data indicate that the anti-phage activity of the lambda RexAB system relies on RexB’s ability to form ion channels, resulting in membrane depolarization, loss of ATP, and termination of synthesis processes, which ultimately lead to infection abortion^29,34^. RexA is supposed to act as a phage infection sensor that, after detecting phage proteins or protein-DNA complexes, binds to and activates the RexB ion channel^29^. Data obtained in this study indicate that the *P. putida* P1-encoded RexA_pp_ and RexB_pp_ have analogous functions, with RexB_pp_ acting as a cell growth-suppressing effector and RexA_pp_ being needed for its triggering. The potential cytotoxic activity of the RexB_pp_ is normally muted in *P. putida*. However, RexA_pp_ overexpression results in the growth suppression of the RexB_pp_-encoding bacteria, whereas the RexB_pp_-deficient strain is not affected by the high RexA_pp_ expression (Fig. 7A, B). Although this RexA_pp_-triggered activation of RexB_pp_ occurred without phage infection, it provides clues on how the *P. putida* RexAB_pp_ system may also function under phage infection conditions. We propose that, similar to the lambda Rex system, the antiphage activity of *P. putida* RexAB_pp_ results from the RexB_pp_-caused loss of membrane integrity. This is supported by increased membrane permeability to SYTOX Green and propidium iodide, as well as leakage of cytoplasmic enzyme β-galactosidase from RexB_pp_-triggered bacteria (Fig. 7E, F, H). However, it is essential to note that these effects were observed only in a small subpopulation of bacteria, indicating that despite severe growth suppression, most bacteria retained overall cell integrity and did not die. The mechanism by which RexB_pp_ is activated by RexA_pp_ most likely involves the binding of two proteins. Although the two-hybrid analysis with fusion proteins did not prove binding between RexA_pp_ and RexB_pp_ (Fig. 5), this does not exclude the possibility that interaction occurs between native proteins or under certain conditions. We hypothesize that under normal growth conditions, RexA_pp_ and RexB_pp_ do not interact, and the toxicity of RexB_pp_ is not expressed. However, RexA_pp_ overexpression or activation by phage infection results in RexA_pp_ and RexB_pp_ interaction, triggering the RexB_pp_-mediated membrane damage. Our data align with observations on the behavior of the lambda RexAB proteins, where RexA overexpression can also activate the RexB channels in uninfected cells, leading to a loss of membrane integrity^54^. It has been shown that the anti-phage ability of the lambda RexAB system is sensitive to the stoichiometry of the two proteins, and overexpression of either RexA or RexB nullifies the phage exclusion ability of the system^29,55^. Our data suggest that the correct stoichiometry of RexA_pp_ and RexB_pp_ is also important for the anti-phage activity of the *P. putida* RexAB_pp_ system, as the reintroduction of the *rexA*_*pp*_ gene into the Δ*rexA*_*pp*_ strain did not restore the phage defense.

To date, only five RexAB-like systems have been described, including the *P. putida* P1-encoded RexAB_pp_ identified in the current study. Three systems, RexAB_pp_, CarolAnn-encoded gp44/gp43^37^, and Sbash-encoded gp30/gp31^38^ resemble the lambda Rex exclusion system in many aspects: they require both RexA and RexB counterparts for antiphage activity, and the RexB membrane protein acts as a defense effector, while RexA stimulates its activity, leading to the impairment of membrane integrity and growth suppression. Whether RexB activation is accompanied by cell death, as is usually proposed^29,37,38^, remains unclear, as lambda lysogens infected with T4 *rII* have been shown to survive after a temporary growth arrest^36^. Our data are more consistent with a model of RexB_pp_ causing phage abortion due to growth suppression rather than cell death, because the RexB_pp_-inhibited bacteria can still form very slowly growing colonies on the medium (Fig. 7B).

The fifth Rex-like phage defense system described to date, the mycobacteriophage Butters-encoded gp30/gp31, differs essentially from other Rex systems^39^. In the Butters defense system, the antiphage effector is the cytosolic Gp30 (RexA-like protein), while the membrane-located Gp31 (RexB-like protein) is not required for defense. The role of Butters-encoded Gp31 appears to be the binding and sequestration of the cytotoxic activity of Gp30 under normal conditions^39^. Thus, while the antiviral effect of lambda RexAB results from the RexA binding and activating RexB, the roles are reversed in Butters’ system – the antiviral activity occurs after RexA is liberated from the RexB interaction.

RexAB-type phage defense systems also differ in the way they are activated during phage infection. While not demonstrated, it has been proposed that lambda RexA is activated by phage recombination or replication intermediates^34^. The isolation of defense escape mutants showed that the Rex systems of the CarolAnn and Sbash phages are triggered by different proteins of the infecting phage: the toxicity of the CarolAnn gp44/gp43 is promoted by a Kita phage protein Gp53^37^, and the Sbash system is activated by phage Crossroads genes gp132 and gp141^38^. Despite several efforts, we were unable to isolate phage mutants that could evade the RexAB_pp_ defense, indicating that the phage component triggering the RexAB_pp_ system is most likely essential for phage lytic growth and therefore cannot be mutated. Given that RexA_pp_ is a DNA-binding protein, one can hypothesize that it possibly senses the phage replication intermediate DNA–protein complexes, as is proposed for lambda RexA^34^.

The cryptic prophages in the *P. putida* genome impose a fitness cost on the bacterium under conditions of DNA damage^41^ and during intraspecific competition in the rhizosphere^44^. Current work suggests that the costs associated with prophage carriage can be offset by the benefits provided by the presence of phage defense genes. This is likely the reason why the cryptic prophages have been stably maintained in the genome throughout evolution.

## Material and methods

Bacterial strains, plasmids, and growth conditions. The bacterial strains and plasmids used are listed in Supplementary Table 1. All strains constructed in this study are derivatives of *P. putida* PaW85^56^, which is isogenic to KT2440^57^. Bacteria were grown in lysogeny broth (LB). If selection was necessary, the growth medium was supplemented with kanamycin (50 µg mL^−1^), ampicillin (100 µg mL^−1^), or gentamycin (10 µg mL^−1^) for *E. coli*, and benzylpenicillin (1500 µg mL^−1^), kanamycin (50 µg mL^−1^), or gentamycin (10 µg mL^−1^) for *P. putida*. *E. coli* was incubated at 37 °C and *P. putida* at 30 °C, except for phage infection experiments, which were conducted at 20 °C. Bacteria were electrotransformed according to the protocol of Sharma and Schimke^58^.

### Construction of strains and plasmids

The *P. putida* PaW85 deletion strains were constructed using a homologous recombination-based protocol that generates scarless deletions^59,60^. The pEMG- and pSNW2-based plasmids containing deletion loci were created by joining the upstream and downstream regions of the DNA fragment intended to be deleted by overlap extension PCR, followed by restriction cloning. Plasmids generated are listed in Supplementary Table 1, and oligonucleotides used in PCR amplifications are listed in Supplementary Table 2. For the inducible expression of HicB2 antitoxin and HicB2 toxin, the PCR-amplified *hicB2* was cloned under the control of LacI-repressed *tac* promoter in plasmid pBRlacItac, and PCR-amplified *hicA2* under the control of arabinose-inducible P_BAD_ promoter in plasmid pBAD33 (Supplementary Table 2). Expression plasmids for two-hybrid analysis were constructed by fusing RexA_pp_ or RexB_pp_ to either end of T18 and T25 fragments of CyaA adenylate cyclase by employing Gibson assembly. The primers used are listed in Supplementary Table 2. For protein purification, the expression plasmid with C-terminally His_6_-tagged RexA_pp_ was engineered by using oligonucleotides listed in Supplementary Table 2 and fragment joining by Gibson assembly. Transcriptional fusion of PP_3896 with *lacZ* was constructed by restriction cloning of a PCR-generated promoter-containing fragment into plasmid p9TT_B_lacZ. For the inducible expression of RexA_pp_, the *rexA*_*pp*_ was amplified from the *P. putida* PaW85 genome and cloned under the control of LacI-repressed *tac* promoter in plasmid pSEVA/lacItac. For delivering the *lacI*^q^*-P*_*tac*_*-rexA*_*pp*_ expression cassette into *P. putida* genome, the cassette was subcloned from pSEVA-lacItac-rexA_pp_ into pGP-miniTn7-ΩGm as a NotI-cleaved fragment. For the complementation of the ΔP1 strain with PP_3896, the PCR-amplified PP_3896 with its native promoter (Supplementary Table 2) was cloned into pGP-miniTn7-ΩGm as a NotI/KpnI-cleaved fragment. For the construction of Δ*rexA*_*pp*_-tac-*rexA*_*pp*_, Δ*rexAB*_*pp*_-tac-*rexA*_*pp*_, Δ*rexA*_*pp*_*Gm*, Δ*rexAB*_*pp*_*Gm,* and ΔP1/3896 strains, the miniTn7 delivery plasmid pGPTn7Gm-lacItac-rexA_pp_ or pGP-miniTn7-ΩGm or pGPTn7Gm-3896 was coelectroporated together with puXBF13 helper plasmid into Δ*rexA*_*pp*_ or Δ*rexAB*_*pp*_ or ΔP1 strains, gentamycin-resistant bacteria were selected, and the miniTn7 insertion into the *glmS* locus was verified by PCR.

### Plaque assays

Bacteria were grown in LB medium overnight at 20 °C. The bacterial cultures were diluted 15-fold into fresh LB medium and grown until OD_580_∼1 at 20 °C. Next, 200 µL of the bacterial culture was mixed with 5 mL of melted 0.3% LB agar medium (42 °C) containing 10 mM CaCl_2_ and overlaid on 1.5% LB agar plates containing 0.03 µg/mL ciprofloxacin to create a bacterial lawn. 1.5 µL drops of ten-fold dilutions of phage lysates were spotted on the bacterial lawns. Plates were incubated overnight at 20 °C. The formation of plaques was assessed.

### Kill/rescue assay

To conduct the kill/rescue assay, pBRlacItac-hicB2 and pBAD33-hicA2 were co-electroporated into *E. coli* DH5α competent cells. Single colonies containing both plasmids were selected and grown overnight in LB liquid medium supplemented with 20 μg/mL chloramphenicol and 100 μg/mL ampicillin to select for the plasmids, 5 mM IPTG to induce the antitoxin expression, and 0.2% glycerol to repress toxin expression. The optical densities of overnight-grown cultures were measured, and the bacteria were diluted into fresh medium to an OD_580_ of 0.1. The LB growth medium was always supplemented with chloramphenicol and ampicillin, but could additionally contain either 5 mM IPTG for the induction of antitoxin expression, 10 mM arabinose for the induction of toxin expression, or both IPTG and arabinose for the induction of expression of both genes. The bacterial cultures were grown on a microtiter plate at 400 rpm at 30 °C, and the optical densities of the cultures were measured using a POLARstar Omega plate reader every 14 min.

### qPCR

For the qPCR assay, bacteria were grown for approximately 18 h in LB medium or LB medium supplemented with 0.03 µg/mL ciprofloxacin to induce DNA stress. The genomic DNA was isolated with GeneJET Genomic DNA Purification Kit (Thermo Fisher Scientific) according to the manufacturer’s protocol.

The excision frequency of prophage P1 from the *P. putida* genome was analyzed with primers flanking the P1 locus (Supplementary Table 2). The extrachromosomal P1 circle was determined using primers specific to both P1 ends (Supplementary Table 2), and the P1 copy number was assessed with primers specific to a DNA region within the P1 (Supplementary Table 2). *rpoD* was used as the reference gene. The qPCR assay was performed on the Rotor-Gene Q system (QIAGEN) using the Maxima SYBR Green/ROX qPCR Master Mix (2X) (Thermo Fisher Scientific) according to the manufacturer’s protocol. 1 or 10 ng of genomic DNA was used for each reaction. Raw data were analyzed using the Rotor-Gene Q software v. 2.02 (QIAGEN), and DNA amounts were calculated with LinRegPCR software v. 2013.0^61^. Data from at least five independent biological replicates were averaged and normalized against *rpoD* levels.

### Bioinformatic analysis

Preliminary data on the PP_5643 and PP_5644 genes was obtained from the Pseudomonas Genome Database^46^. The transmembrane topology of PP_5644 was analyzed with DeepTMHMM^48^. Sequence homology searches were performed using NCBI BLAST (https://blast.ncbi.nlm.nih.gov/Blast.cgi) and HHpred^62,63^. The AlphaFold 3 server^64^ was used to generate protein structure models, and UCSF ChimeraX^65^ was used to visualize the results. Structural homology searches and alignments were performed in the DALI server^66^.

### Protein purification

For RexA_pp_-his_6_ purification, *E. coli* BL21(DE3) cells carrying the pET-rexA-His plasmid (Supplementary Table 2) were pre-grown overnight in liquid LB medium containing ampicillin at 37 °C and then diluted into 500 mL of LB medium containing ampicillin to an initial optical density at 580 nm (OD_580_) of approximately 0.1. Bacteria were grown at 37 °C until OD_580_ of approximately 1, after which the temperature was lowered to 20 °C and IPTG (final concentration of 0.5 mM) was added to induce RexA_pp_-His_6_ production. After overnight induction, cells were pelleted and stored at − 80 °C. For protein purification, cells were thawed and resuspended in lysis buffer (20 mM HEPES, pH 7.4, 500 mM NaCl, 5% glycerol, 5 mM β-mercaptoethanol, 5 mM MgCl_2_, 1 mM PMSF, DNase I (10 U), and lysozyme (2 mg)) before sonication. The lysate was cleared by centrifugation, filtered through a 0.45 µm filter, and loaded onto a 1 mL His-Trap HP (Cytiva Life Sciences) column, which was equilibrated with buffer A (20 mM HEPES, pH 7.4, 500 mM NaCl, 5% glycerol, 5 mM β-mercaptoethanol). Protein purification was performed by fast protein liquid chromatography (FPLC) using an ÄKTA go™ chromatography system (Cytiva Life Sciences). Proteins were eluted using a linear 50–500 mM imidazole gradient. Pooled fractions were concentrated using an Ultracell 30 kDa cut-off centrifugal filter and transferred to a Superdex 75 Increase 10/300 GL (Cytiva Life Sciences) SEC Column, equilibrated in buffer A. Fractions containing RexA_pp_-His_6_ were collected.

### Electrophoretic mobility shift assay (EMSA)

For the EMSA assay, two different PCR fragments were amplified from the *P. putida* PaW85 genome: fragment 1 (175 bp, positions 4,415,629 to 4,415,799 in the *P. putida* genome) and fragment 2 (272 bp, positions 4,411,510 to 4,411,770 in the *P. putida* genome) (Supplementary Table 2). Fragment 1 was FAM-labeled, and fragment 2 was unlabeled. The PCR fragments were purified using the MicroElute® Cycle-Pure & Gel Extraction Kit (Omega Bio-tek).

Each EMSA reaction contained 1 × Binding Buffer (20 mM Tris–HCl, pH 8.0, 50 mM NaCl, 5% glycerol), 0.6 µM FAM-labeled DNA fragment, and the indicated concentration of RexA_pp_-His_6_. Reactions were incubated for 30 min at room temperature. When the FAM-labeled DNA probe was used, the reactions were run on a 5% polyacrylamide gel in 0.5 × Tris–Borate-EDTA (TBE) buffer (120 V, 45 min) at 4 °C, and the gel was visualized using Phosphorimager Typhoon RGB. When an unlabeled DNA probe was used, the reactions were run on a 1% agarose gel supplemented with 0.5 μg/mL ethidium bromide in Tris–acetate-EDTA (TAE) buffer (120 V, 20 min) at 4 °C and visualized under UV light.

### Bacterial two-hybrid assay (BACTH)

The adenylate cyclase-deficient *E. coli* reporter strain BTH101 was co-electroporated with two recombinant plasmids encoding hybrid proteins in which the proteins of interest were fused with either T25 or T18, i.e., fragments of *Bordetella pertussis* adenylate cyclase^49^. Co-transformants were grown for 1 h in LB medium, then tenfold serially diluted and spotted on LB agar plates supplemented with 0.5 mM IPTG, X-gal (100 μg/mL), and antibiotics kanamycin and ampicillin. Plates were incubated at 30 °C for up to 72 h before imaging.

### Tests for determining the RexB_pp_-mediated toxicity effects

For growth curves, bacteria were grown overnight in liquid LB medium supplemented with gentamycin. The optical density of overnight cultures was measured, and the bacteria were diluted into LB supplemented with gentamycin to an OD_580_ of 0.1. For RexA_pp_ induction from the tac-*rexA*_*pp*_ expression cassette, 0.5 mM IPTG was added to the growth medium. Cells were grown without shaking on a microtiter plate at 30 °C, and the optical density (OD) at 580 nm was measured every 10 min using a TECAN Sunrise™ microplate reader.

For assaying the bacterial growth on solid medium, overnight-grown bacteria in LB medium supplemented with gentamycin were diluted tenfold, and 5 μL drops of the dilutions were spotted on LB plates supplemented with gentamycin and, if indicated, with 0.5 mM IPTG for RexA_pp_ induction from tac-*rexA*_*pp*_ expression cassette. Plates were incubated at 30 °C and imaged at 24, 48, and 72 h. After 72 h, Δ*rexA*_*pp*_-tac-*rexA*_*pp*_ mutants that emerged on plates supplemented with 0.5 mM IPTG were picked and analyzed with PCR and sequencing.

For the β-galactosidase leakage assay, the Δ*rexA*_*pp*_-tac-*rexA*_*pp*_ and Δ*rexAB*_*pp*_-tac-*rexA*_*pp*_ strains containing the p9TT_B_lacZ/amrZ plasmid were grown overnight in 5 mL LB medium. β-Galactosidase activity was determined both from the cells and from the cell-free supernatant^67^. The abundance of the enzyme in the supernatant was expressed as a percentage of the total enzyme activity measured from the cells.

### Flow cytometry analysis

Bacteria were grown overnight in 5 mL LB medium supplemented with gentamycin. After the 50-fold dilution into fresh 5 mL LB medium, the bacteria were grown for 1.5 h at 30 °C. Then, 0.5 mM IPTG was added to the medium to induce the RexA_pp_ expression from the tac-*rexA*_*pp*_ cassette, and bacteria were further grown for 3.5 h. The optical density at 580 nm was measured, and the cell suspensions were diluted with PBS to an OD_580_ of 0.03. For staining of bacteria, the diluted cell suspensions were supplemented with different fluorescent dyes (all obtained from Invitrogen) in the following final concentrations: 5 μM SYTO 9, 50 μM propidium iodide (PI), 0.8 μM SYTOX Green, or 5 μM DiSC_3_(5). Samples were incubated at 22 °C in the dark for 20 min, and approximately 30,000 events from every sample were analyzed with the FACSAria I flow cytometer (BD Biosciences). Samples stained with SYTO 9, PI, or SYTOX Green were excited with a 488 nm laser beam. The emission signals were detected using an optical filter with a 530/30 bandpass for SYTO 9 and SYTOX Green, and a filter with a 616/23 bandpass for PI. Samples stained with DiSC_3_(5) were excited with a 633 nm laser beam, and the emission signals were detected using an optical filter with a 660/20 bandpass. Data were analysed using FlowJo software. Populations of intact and PI-permeable cells were defined as previously^68^.

## Fundings

This work was supported by the Estonian Research Council (grant PRG1431 to RH, and STP39 to AA) and by the European Union (ERC-StG, PhaBacArms, grant no 101116205 to HT; Views and opinions expressed are, however, those of the author(s) only and do not necessarily reflect those of the European Union or the European Research Council Executive Agency. Neither the European Union nor the granting authority can be held responsible for them).

## Supplementary Information

Below is the link to the electronic supplementary material.

Supplementary Material 1

## Data Availability

Summarized and analyzed data supporting the findings of this study are available within the paper and its Supplementary Information. All datasets generated and/or analyzed during this study are available from the corresponding author on reasonable request.
