# Supplementary material for: Prophage-encoded RexAB-type phage defense system in Pseudomonas putida
Source: Sci Rep. 2026 Jan 21;16:5898. doi: 10.1038/s41598-026-36734-5 (PMC12894663; doi:10.1038/s41598-026-36734-5)
Supplement: Supplementary file 1 — Supplementary Material 1 [file 41598_2026_36734_MOESM1_ESM.pdf]

Prophage-encoded RexAB-type phage defense system in *Pseudomonas putida*

Sirli Rosendahl[a], Anu Kängsep[a], Andres Ainelo[a], Anita Lipu[a], Hedvig Tamman[a], Rita Hõrak[a*]

[a] Institute of Molecular and Cell Biology, University of Tartu, Tartu, Estonia*

Address correspondence to Rita Hõrak, rita.horak@ut.ee

Supplementary Table 1. Strains and plasmids.

Strain or plasmid	Genotype or characteristics	Source or reference
Escherichia coli		
DH5α	*supE44* Δ*lacU169* (ϕ80 *lacZ*ΔM15) *recA1 endA1 hsdR17 thi-1 gyrA96 relA1*	Lab collection
DH5αλpir	λpir lysogen of DH5α	[1]
BTH101	*F-,cya-99, araD139, galE15, galK16, rpsL1 (Str^r), hsdR2, mcrA1, mcrB1*	[2]
BL21(DE3)	hsdS gal (λcI ts857 ind-1 Sam7 nin-5 lacUV5-T7 gene 1)	[3]
Pseudomonas putida		
PaW85	Wild-type, isogenic to KT2440	[4]
ΔP1	PaW85 with spontaneous deletion of prophage P1	[5]
ΔP2	PaW85 with deletion of prophage P2	This study
ΔP3	PaW85 with deletion of prophage P3	This study
ΔP4	PaW85 with deletion of prophage P4	This study
Δ4ϕ	PaW85 with deletion of prophages P1, P2, P3, and P4	[5]
Δ*hicA2*	PaW85 with deletion of P1-encoded *hicA2* toxin gene (PP_3900)	This study
Δ*hicAB2*	PaW85 with deletion of P1-encoded *hicAB2* TA operon (PP_3900-3899)	This study
P1ΔL39	39 genes (PP_3849-PP_5641) are deleted from prophage P1 left arm	This study
P1ΔR21	21 genes (PP_3900-PP_3917) are deleted from prophage P1 right arm	This study
P1ΔC9	9 genes (PP_3886-PP_3894) are deleted from prophage P1 central region	This study
P1ΔC4	PP_3897, PP_3898, PP5643 and PP_5644 are deleted from prophage P1	This study
Δ3897	PP_3897 is deleted from prophage P1	This study
Δ3898	PP_3898 is deleted from prophage P1	This study
Δ5643 (Δ$rexA_{pp}$)	PP_5643 ($rexA_{pp}$) is deleted from prophage P1	This study
Δ5644 (Δ$rexB_{pp}$)	PP_5644 ($rexB_{pp}$) is deleted from prophage P1	This study
Δ5643-5644 (Δ$rexAB_{pp}$)	PP_5643 and PP_5644 ($rexAB_{pp}$) are deleted from prophage P1	This study
Δ$rexA_{pp}$-tac-$rexA_{pp}$	Δ$rexA_{pp}$ with $lacI^q$-P_{tac}-$rexA_{pp}$ expression cassette in the *glmS* locus (Gm^r)	This study
Δ$rexAB_{pp}$-tac-$rexA_{pp}$	Δ$rexAB_{pp}$ with $lacI^q$-P_{tac}-$rexA_{pp}$ expression cassette in the *glmS* locus (Gm^r)	This study
Δ$rexA_{pp}Gm$	Δ$rexA_{pp}$ with mini-Tn*7*-ΩGm in the *glmS* locus	This study
Δ$rexAB_{pp}Gm$	Δ$rexAB_{pp}$ with mini-Tn*7*-ΩGm in the *glmS* locus	This study
ΔP1/3896	ΔP1 with PP_3896 in the *glmS* locus (Gm^r)	This study
Plasmids		
pEMG	Plasmid for homologous recombination, *lacZa* with two flanking I-SceI sites (Km^r)	[6]
pSW (I-SceI)	Plasmid coding for I-SceI endonuclease for allelic exchange experiments (Bp^r)	[7]
pEMG-R2	Plasmid containing a chimeric DNA fragment for deleting prophage P2 (Km^r)	[8]
pEMG-R3	Plasmid containing a chimeric DNA fragment for deleting prophage P3 (Km^r)	[8]
pEMG-P4	Plasmid containing a chimeric DNA fragment for deleting prophage P4 (Km^r)	[8]
pBRlacItac	Expression vector containing $lacI^q$ repressor-controlled P_{tac} promoter (Ap^r)	[9]
pBRlacItac-hicB2	Plasmid for antitoxin HicB2 inducible expression (Ap^r)	This study
pBAD33	Expression vector containing arabinose-controlled P_{BAD} promoter (Cm^r)	[10]
pBAD33-hicA2	Plasmid for toxin HicA2 inducible expression (Cm^r)	This study
pEMG-ΔhicB2	pEMG containing a chimeric DNA fragment for deleting *hicB2* (PP_3900) (Km^r)	This study
pEMG-ΔhicBA2	pEMG containing a chimeric DNA fragment for deleting *hicBA2* (PP_3900-PP_3899) (Km^r)	This study
pEMG-ΔL39	pEMG containing a chimeric DNA fragment for deleting 39 genes (PP_3849-PP_5641) from P1 (Km^r)	This study
pEMG-ΔR21	pEMG containing a chimeric DNA fragment for deleting 21 genes (PP_3900-PP_3917) from P1 (Km^r)	This study
pEMG-ΔC9	pEMG containing a chimeric DNA fragment for deleting 9 genes (PP_3886-PP_3894) from P1 (Km^r)	This study
pEMG-ΔC4	pEMG containing a chimeric DNA fragment for deleting PP_3897, PP_3898, PP5643 and PP_5644 from P1 (Km^r)	This study
pSNW2	pEMG derivate with P14g(BCD2)→msfGFP (Kmr)	[11]
pSNW2-Δ3897	pSNW2 containing a chimeric DNA fragment for deleting PP_3897 (Km^r)	This study
pSNW2-Δ3898	pSNW2 containing a chimeric DNA fragment for deleting PP_3898 (Km^r)	This study
pSNW2-Δ$rexA_{pp}$	pSNW2 containing a chimeric DNA fragment for deleting PP_5643 (Km^r)	This study
pSNW2-Δ$rexB_{pp}$	pSNW2 containing a chimeric DNA fragment for deleting PP_5644 (Km^r)	This study
pSNW2-Δ$rexAB_{pp}$	pSNW2 containing a chimeric DNA fragment for deleting PP_5643-5644 (Km^r)	This study
pT25	Plasmid encoding the T25 fragment (1-224 amino acids) of *cyaA* (Km^r)	[2]
pT18	Plasmid encoding the T18 fragment (225-399 amino acids) of *cyaA* (Ap^r)	[2]
pT25-graT	Plasmid encoding the T25 fragment fused with *graT* (Km^r)	[12]
pT18-graA	Plasmid encoding the T18 fragment fused with *graA* (Ap^r)	[12]

pT25-Ct-rexA	Plasmid encoding the *cyaA(T25)-rexA*$_{pp}$ fusion (Km^r)	This study
pT25-Nt-rexA	Plasmid encoding the *rexA*$_{pp}$*-cyaA(T25)* fusion (Km^r)	This study
pT25-Ct-rexB	Plasmid encoding the *cyaA(T25)-rexB*$_{pp}$ fusion (Km^r)	This study
pT25-Nt-rexB	Plasmid encoding the *rexB*$_{pp}$*-cyaA(T25)* fusion (Km^r)	This study
pT18-Ct-rexA	Plasmid encoding the *cyaA(T18)-rexA*$_{pp}$ fusion (Ap^r)	This study
pT18-Nt-rexA	Plasmid encoding the *rexA*$_{pp}$*-cyaA(T18)* fusion (Ap^r)	This study
pT18-Ct-rexB	Plasmid encoding the *cyaA(T18)-rexB*$_{pp}$ fusion (Ap^r)	This study
pT18-Nt-rexB	Plasmid encoding the *rexB*$_{pp}$*-cyaA(T18)* fusion (Ap^r)	This study
pET11c	Protein expression vector	Lab collection
pET-rexA-His	pET11c for expression of RexA$_{pp}$ with C-terminal His$_6$ tag (Ap^r)	This study
pSEVA/lacItac	Expression vector containing *lacI*^{q} repressor-controlled *P*$_{tac}$ promoter (Km^r)	[13]
pSEVA-lacItac-rexA$_{pp}$	Plasmid with *lacI*^{q}-*P*$_{tac}$-*rexA*$_{pp}$ expression cassette (Km^r)	This study
pGP-miniTn7-ΩGm	pGP704L-based delivery plasmid for mini-Tn*7*-ΩGm (Ap^r, Gm^r)	[14]
pGPTn7Gm-lacItac-rexA$_{pp}$	pGP-miniTn7-ΩGm with *lacI*^{q}-*P*$_{tac}$-*rexA*$_{pp}$ expression cassette (Ap^r, Gm^r)	This study
pGPTn7Gm-3896	pGP-miniTn7-ΩGm with PP_3896 (Ap^r, Gm^r)	This study
pUXBF13	Plasmid coding for the Tn*7* transposition proteins (Ap^r, *mob*$^+$)	[15]
p9TT$_B$lacZ	Promoter probe plasmid with *lacZ* reporter (Ap^r Cm^r)	[16]
p9TT$_B$3896-lacZ	Transcriptional fusion of PP_3896 with *lacZ* in p9TT$_B$lacZ (Ap^r Cm^r)	This study
p9TT$_B$lacZ/amrZ	Transcriptional fusion of PP_4470 with *lacZ* in p9TT$_B$lacZ (Ap^r Cm^r)	[17]

1 Martinez-Garcia, E. & de Lorenzo, V. Engineering multiple genomic deletions in Gram-negative bacteria: analysis of the multi-resistant antibiotic profile of *Pseudomonas putida* KT2440. *Environmental microbiology* **13**, 2702-2716 (2011).

2 Karimova, G., Pidoux, J., Ullmann, A. & Ladant, D. A bacterial two-hybrid system based on a reconstituted signal transduction pathway. *Proceedings of the National Academy of Sciences of the United States of America* **95**, 5752-5756 (1998).

3 Studier, F. W. & Moffatt, B. A. Use of bacteriophage T7 RNA polymerase to direct selective high-level expression of cloned genes. *Journal of molecular biology* **189**, 113-130. (1986).

4 Bayley, S. A. *et al.* Two modes of loss of the Tol function from *Pseudomonas putida* mt-2. *Molecular & general genetics : MGG* **154**, 203-204 (1977). https://doi.org:10.1007/bf00330838

5 Brauer, A. *et al.* Isolation and characterization of a phage collection against Pseudomonas putida. *Environmental microbiology* **26**, e16671 (2024). https://doi.org:10.1111/1462-2920.16671

6 Martinez-Garcia, E. & de Lorenzo, V. Engineering multiple genomic deletions in Gram-negative bacteria: analysis of the multi-resistant antibiotic profile of *Pseudomonas putida* KT2440. *Environmental Microbiology* **13**, 2702-2716 (2011). https://doi.org:10.1111/j.1462-2920.2011.02538.x

7 Wong, S. M. & Mekalanos, J. J. Genetic footprinting with *mariner*-based transposition in *Pseudomonas aeruginosa*. *Proceedings of the National Academy of Sciences of the United States of America* **97**, 10191-10196 (2000). https://doi.org:10.1073/pnas.97.18.10191

8 Martinez-Garcia, E., Jatsenko, T., Kivisaar, M. & de Lorenzo, V. Freeing *Pseudomonas putida* KT2440 of its proviral load strengthens endurance to environmental stresses. *Environmental microbiology* **17**, 76-90 (2015). https://doi.org:10.1111/1462-2920.12492

9 Ojangu, E. L., Tover, A., Teras, R. & Kivisaar, M. Effects of combination of different-10 hexamers and downstream sequences on stationary-phase-specific sigma factor sigma(S)-dependent transcription in *Pseudomonas putida*. *J Bacteriol* **182**, 6707-6713 (2000). https://doi.org:10.1128/jb.182.23.6707-6713.2000

10 Guzman, L. M., Belin, D., Carson, M. J. & Beckwith, J. Tight regulation, modulation, and high-level expression by vectors containing the arabinose PBAD promoter. *Journal of bacteriology* **177**, 4121-4130 (1995). https://doi.org:10.1128/jb.177.14.4121-4130.1995

11 Volke, D. C., Friis, L., Wirth, N. T., Turlin, J. & Nikel, P. I. Synthetic control of plasmid replication enables target- and self-curing of vectors and expedites genome engineering of Pseudomonas putida. *Metab Eng Commun* **10**, e00126 (2020). https://doi.org:10.1016/j.mec.2020.e00126

12 Rosendahl, S., Ainelo, A. & Hõrak, R. The Disordered C-Terminus of the Chaperone DnaK Increases the Competitive Fitness of *Pseudomonas putida* and Facilitates the Toxicity of GraT. *Microorganisms* **9** (2021). https://doi.org:10.3390/microorganisms9020375

13 Tagel, M. *et al.* Pseudouridines of tRNA Anticodon Stem-Loop Have Unexpected Role in Mutagenesis in Pseudomonas sp. *Microorganisms* **9** (2020). https://doi.org:10.3390/microorganisms9010025

14 Jakovleva, J. *et al.* Fis regulates the competitiveness of Pseudomonas putida on barley roots by inducing biofilm formation. *Microbiology (Reading)* **158**, 708-720 (2012). https://doi.org:10.1099/mic.0.053355-0

15 Bao, Y., Lies, D. P., Fu, H. & Roberts, G. P. An improved Tn7-based system for the single-copy insertion of cloned genes into chromosomes of gram-negative bacteria. *Gene* **109**, 167-168 (1991).

16 Kivistik, P. A. *et al.* The ColRS two-component system regulates membrane functions and protects *Pseudomonas putida* against phenol. *Journal of bacteriology* **188**, 8109-8117 (2006).

17 Mumm, K., Ainsaar, K., Kasvandik, S., Tenson, T. & Hõrak, R. Responses of *Pseudomonas putida* to Zinc Excess Determined at the Proteome Level: Pathways Dependent and Independent of ColRS. *J Prot Res* **15**, 4349-4368 (2016). https://doi.org:10.1021/acs.jproteome.6b00420

Supplementary Table 2. Oligonucleotides

Name	Sequence (5'-3')[a]	Usage (cloning method)[b]
3899EcoRI	cggaattctacatagccgccctgg	construction of pBRlacItac-hicB2, pEMG-ΔhicB2, pEMG-ΔhicBA2 and pEMG-ΔR21 (RC)
3900Kpn	aatggtacctaactggaatgattgacc	construction of pBRlacItac-hicB2 and pBAD33-hicA2 (RC)
3900Hindend	ctgaagcttgccgaataggcaatt	construction of pBAD33-hicA2 (RC)
3900del	aagcaggaggattgatctga	construction of pEMG-ΔhicB2 and pEMG-ΔR21 (RC)
3900delpikk	tcagatcaatcctcctgcttcttgcttagacatttgggc	construction of pEMG-ΔhicB2 (RC)
3901Bam	gtggatccggtgatggagcgagt	construction of pEMG-ΔhicB2 and pEMG-ΔhicBA2 (RC)
3899TAdelpikk	ctgtcatagcatcgcaactagggaagcggatgagcct	construction of pEMG-ΔhicBA2 (RC)
3900start	tagttgcgatgctatgacag	construction of pEMG-ΔhicBA2 (RC)
5636_Kpn	ataggtaccggcagcgactgaggat	construction of pEMG-ΔL39 (RC)
P1circ-L	atcgacccttgtagaatgcg	construction of pEMG-ΔL39 (RC)
P1delLpikk	cgcattctacaagggtcgatctgagcatctaacgtctc	construction of pEMG-ΔL39 (RC)
P1delLXba	tttctagatggttcatgatcgagag	construction of pEMG-ΔL39 (RC)
P1delR2	tcagatcaatcctcctgcttcatcacactgagtagagagc	construction of pEMG-ΔR21 (RC)
3920eesXba	tttctagatttcggtttccatgggat	construction of pEMG-ΔR21 (RC)
P1delRKpn	ataggtacctagcggtgagctcgtaga	construction of pEMG-ΔC9 (RC)
P1delkesk	gcgtcgagacgttagatgct	construction of pEMG-ΔC9 (RC)
delkesk2pikk	agcatctaacgtctcgacgcatccattgcagagtgcat	construction of pEMG-ΔC9 (RC)
delkesk2Xba	aatctagagcactatctacaaagaact	construction of pEMG-ΔC9 (RC)
P1delkesk4Bam	aaggatccgtaagcaacgtgatgcc	construction of pEMG-ΔC4 and pSNW2-Δ3897 (RC)
P1delkesk4-pikk	gataggatgagggatagtgttctcgcctttcaagcggca	construction of pEMG-ΔC4 (RC)
3899rev	acactatccctcatcctatc	construction of pEMG-ΔC4 (RC)
P1delkesk4Eco	aagaattcaagaggagtgcttcatag	construction of pEMG-ΔC4 (RC)
P1del3897	gtcatcttccataatttctcg	construction of pSNW2-Δ3897 (RC)
P1del3897-pikk	cgagaaattatggaagatgacgccgattgaagctacc	construction of pSNW2-Δ3897 (RC)
P1del3897Eco	tagaattcgttcgatctgctaatgc	construction of pSNW2-Δ3897 (RC)
P1del3898Eco	atgaattctaccagctcactgtaaaag	construction of pSNW2-Δ3898 (RC)
P1del3898	cttggttagaggcacgggt	construction of pSNW2-Δ3898 (RC)
P1del3898-pikk	acccgtgcctctaaccaaggagtgactcaggatctg	construction of pSNW2-Δ3898 (RC)
P1del3898Bam	aaggat-cctattactacgtgtgcgga	construction of pSNW2-Δ3898 (RC)
P1del5643Bam	aaggatccgattgaagctaccaattg	construction of pSNW2-ΔrexA$_{pp}$ and pSNW2-ΔrexAB$_{pp}$ (RC)
P1del5643	tgtcctgtgcacgaagctttc	construction of pSNW2-ΔrexA$_{pp}$ and pSNW2-ΔrexAB$_{pp}$ (RC)
P1del5643-pikk	aaagcttcgtgcacaggacagcactgatggtgtaacc	construction of pSNW2-ΔrexA$_{pp}$ (RC)
P1del5643Eco	aagaattcgaggaaagggcaatcgc	construction of pSNW2-ΔrexA$_{pp}$ (RC)
P1del5644Bam	aaggatcctaattccgcacgcagct	construction of pSNW2-ΔrexB$_{pp}$ (RC)
P1del5644	tagttggcctatcaacatgg	construction of pSNW2-ΔrexB$_{pp}$ (RC)
P1del5644-pikk	ccatgttgataggccaactaggatataaactacaggag	construction of pSNW2-ΔrexB$_{pp}$ (RC)
P1del5644Eco	aagaattcggcaagcttcagttca	construction of pSNW2-ΔrexB$_{pp}$ and pSNW2-ΔrexAB$_{pp}$ (RC)
P1del43-44-pikk	aaagcttcgtgcacaggacaggatataaactacaggag	construction of pSNW2-ΔrexAB$_{pp}$ (RC)
T25-Ct-Fw	gaggatccccgggtacctaagt	construction of pT25-Ct-rexA and pT25-Ct-rexB (GA)
T25-Ct-Rev	tagagtcgaccctgcagcc	construction of pT25-Ct-rexA and pT25-Ct-rexB (GA)
5643-T25-Ct-Fw	gcagggtcgactctacacaggacagcaacgttcgt	construction of pT25-Ct-rexA (GA)
5643-T25-Ct-Rev	tacccggggatcctcttacaccatcagtgctgtgattgattttattatagac	construction of pT25-Ct-rexA (GA)
5644-T25-Ct-Fw	gcagggtcgactctattgataggccaactagctaaaccttt	construction of pT25-Ct-rexB (GA)
5644-T25-Ct-Rev	tacccggggatcctcctactcctgtagtttatatcctaggtagtaaagcc	construction of pT25-Ct-rexB (GA)
T25-Nt-Fw	accatgcagcaatcgcat	construction of pT25-Nt-rexA and pT25-Nt-rexB (GA)
T25-Nt-Rev	catagctgtttcctgtgtgaaattgttatcc	construction of pT25-Nt-rexA and pT25-Nt-rexB (GA)
5643-T25-Nt-Fw	tcacacaggaaacagctatgcacaggacagcaacgttcgttga	construction of pT25-Nt-rexA (GA)

5643-T25-Nt-Rev	tgatgcgattgctgcatggtcaccatcagtgctgtgattgattttattatagactg	construction of pT25-Nt-rexA (GA)
5644-T25-Nt-Fw	acacaggaaacagctatgttgataggccaactagctaaacct	construction of pT25-Nt-rexB (GA)
5644-T25-Nt-Rev	cgattgctgcatggtctcctgtagtttatatcctaggtagtaaagccc	construction of pT25-Nt-rexB (GA)
T18-Ct-Fw	cactgcaggtcgactctagagg	construction of pT18-Ct-rexA and pT18-Ct-rexB (GA)
T18-Ct-Rev	gcgttccactgcgcc	construction of pT18-Ct-rexA and pT18-Ct-rexB (GA)
5643-T18-Ct-Fw	ggcgcagtggaacgccacaggacagcaacgttcgttga	construction of pT18-Ct-rexA (GA)
5643-T18-Ct-Rev	agtcgacctgcagtgttacaccatcagtgctgtgattgat	construction of pT18-Ct-rexA (GA)
5644-T18-Ct-Fw	cgctgggcgcagtggaacgcttgataggccaactagctaaacctttgg	construction of pT18-Ct-rexB (GA)
5644-T18-Ct-Rev	tctagagtcgacctgcagtgctactcctgtagtttatatcctaggtagtaaagc	construction of pT18-Ct-rexB (GA)
T18-Nt-Fw	accatgattacgccaagcttagc	construction of pT18-Nt-rexA and pT18-Nt-rexB (GA)
T18-Nt-Rev	catagctgtttcctgtgtgaaattgttatcc	construction of pT18-Nt-rexA and pT18-Nt-rexB (GA)
5643-T18-Nt-Fw	acacaggaaacagctatgcacaggacagcaacgttcgttga	construction of pT18-Nt-rexA (GA)
5643-T18-Nt-Rev	cttggcgtaatcatggtcaccatcagtgctgtgattgattttattataga	construction of pT18-Nt-rexA (GA)
5644-T18-Nt-Fw	tcacacaggaaacagctatgttgataggccaactagctaaacct	construction of pT18-Nt-rexB (GA)
5644-T18-Nt-Rev	taagcttggcgtaatcatggtctcctgtagtttatatcctaggtagtaaagccc	construction of pT18-Nt-rexB (GA)
pET-6his-C-Rev	catcatcaccaccatcactaaggatc	construction of pET-rexA-his (GA)
pET-6his-C-Fw	catatgtatatctccttcttaaagttaaacaaaattatttctagagggg	construction of pET-rexA-his (GA)
pet5643-Rev	gtgatggtggtgatgatgcaccatcagtgctgtgattgattttattatagact	construction of pET-rexA-his (GA)
pet5643-Fw	actttaagaaggagatatacatatgcacaggacagcaacgt	construction of pET-rexA-his (GA)
43Pstuus	aaactgcagaacaataaataagtctacaacac	construction of pSEVA-lacItac-rexA$_{pp}$ (RC)
5643Xba	aatctagacccaaaggtttagctagttg	construction of pSEVA-lacItac-rexA$_{pp}$ (RC)
cro_pr_fw_Hind	aaaagcttgaatttgccctcagtca	construction of p9TT$_B$3896-lacZ (RC) and amplification of DNA fragment 2 for EMSA
cro_pr_rev_Hind	aaaagcttccatatcagaagtatcagg	construction of p9TT$_B$3896-lacZ (RC) and amplification of DNA fragment 2 for EMSA
cro_pr_Fw_Not	ttgcggccgcttgaatttgccctcagtca	construction of pGPTn7Gm-3896 (RC)
3896_Rev_Kpn	tttggtacctttcaagcggcagccca	construction of pGPTn7Gm-3896 (RC)
Tn7R109	cagcataactggactgatttcag	verification of miniTn7 insertion in *glmS* locus
Tn7GlmS	aatctggccaagtcggtgac	verification of miniTn7 insertion in *glmS* locus
delP1qFw	agtttcggaactcttgccttt	measuring the P1 excision frequency
delP1qRev	gccgagtagcaaagtggttat	measuring the P1 excision frequency
P1circ-R	caagccttccaaatgatgaagtt	determining the P1 extrachromosomal circle
P1circ-L	atcgacccttgtagaatgcg	determining the P1 extrachromosomal circle
PP_3886qFw	tgacgaattggcgagtgtt	measuring the copy number of P1
PP_3886qRev	cccacgctgactgttacc	measuring the copy number of P1
rpoDqFw	gcaacagcagtctcgtatca	amplification of the *rpoD* gene
rpoDqRev	atgatgtcttccacctgttcc	amplification of the *rpoD* gene
3901FAM	acggtggtagcgctccattt	amplification of DNA fragment 1 (5'-FAM-labelled) for EMSA
3900Hind	gagaagcttttcttgcttagacatt	amplification of DNA fragment 1 (5'-FAM-labelled) for EMSA

[a] The sites of restriction enzymes used in cloning are underlined.

[b] Plasmids were constructed either with restriction cloning (RC) or by Gibson assembly (GA).

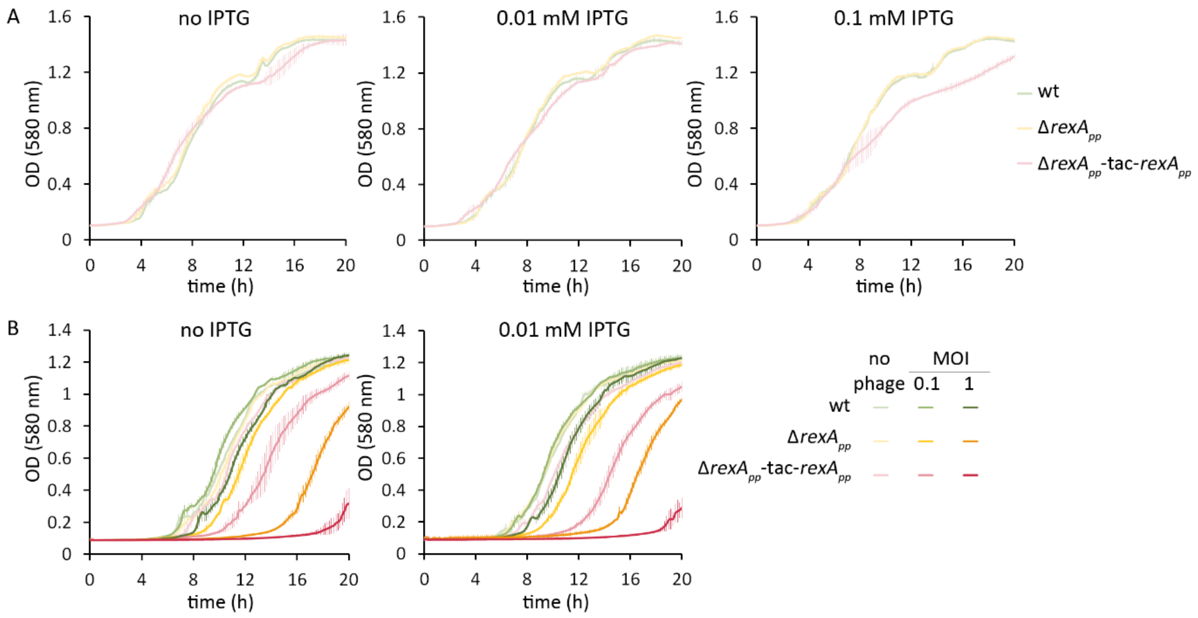

Supplementary Figure S1.

A. Growth curves of *P. putida* wt, Δ*rexA*$_{pp}$, and Δ*rexA*$_{pp}$-tac-*rexA*$_{pp}$ strains in LB liquid medium at 20 °C without IPTG, or with 0.01 mM or 0.1 mM IPTG. Overnight-grown bacterial cultures were diluted 12.5-fold into fresh medium and grown until OD_{580}~1 at 20 °C. Next, the bacterial cultures were diluted into fresh medium to an OD_{580} of 0.1 and grown on a microtiter plate at 20 °C without shaking. If shown, medium was supplemented with either 0.01 mM or 0.1 mM IPTG for $RexA_{pp}$ induction. The optical density at 580 nm was measured every 10 minutes using a BioTek Synergy H1 microplate reader. The average of four technical replicates, along with the standard deviation, is presented.

B. Growth curves of *P. putida* wt, Δ*rexA*$_{pp}$, and Δ*rexA*$_{pp}$-tac-*rexA*$_{pp}$ strains when infected with phage Kurepalu-2 (9A) in LB liquid medium at 20 °C without IPTG or with 0.01 mM IPTG. Overnight-grown bacterial cultures were diluted 12.5-fold into fresh medium and grown until OD_{580}~1 at 20 °C. Next, the bacterial cultures were diluted into fresh medium to an OD_{580} of 0.1. If shown, the medium was supplemented with 0.01 mM IPTG for $RexA_{pp}$ induction, and/or cells were infected with phage Kurepalu-2 at a multiplicity of infection (MOI) of 0.1 or 1. Cultures were grown on a microtiter plate at 20 °C without shaking. The optical density at 580 nm was measured every 10 minutes using a BioTek Synergy H1 microplate reader. The average of three technical replicates, along with the standard deviation, is presented.

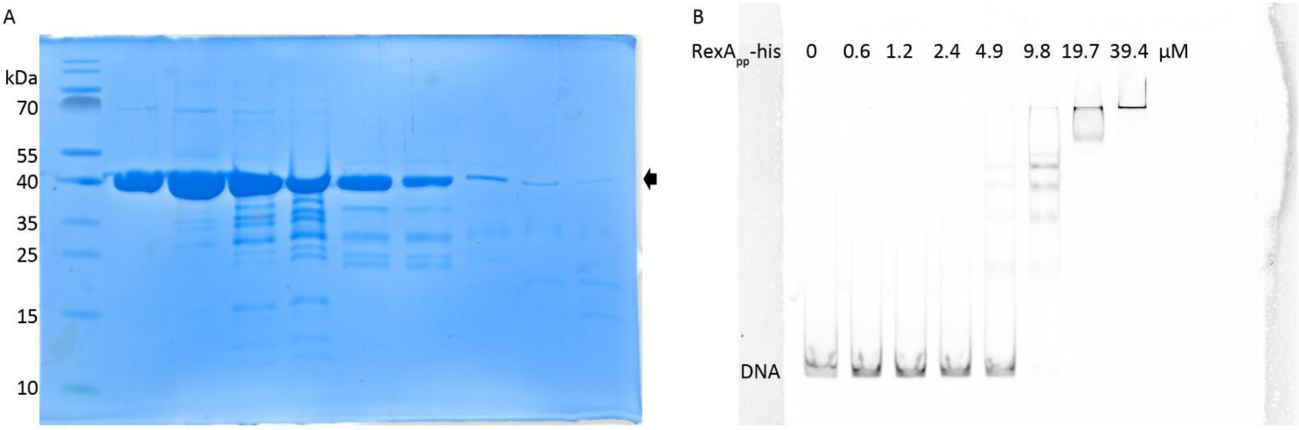

Supplementary Figure S2.

A. Original gel image of Figure 6A without cropping and editing. Collected fractions of RexA$_{pp}$-His$_6$ (Mw 37.5 kDa) after size exclusion chromatography (SEC).

B. Original gel image of Figure 6B without cropping and editing.
